# Prevalence and causes of blindness and distance visual impairment in Chinese adult population in 2022 during the COVID-19 pandemic: a cross-sectional study

**DOI:** 10.1038/s41598-024-54325-0

**Published:** 2024-02-16

**Authors:** Hua Wang, Zhi Xu, Dandan Chen, Huihui Li, Junyan Zhang, Qinghuai Liu, Han Shen

**Affiliations:** 1 Lianyungang Ophthalmologic Hospital, Lianyungang, 222000 China; 2 Department of Ophthalmology, The First Affiliated Hospital with Nanjing Medical University, Nanjing, 210029 China; 3 Department of Clinical Epidemiology and Evidence-Based Medicine, Shanxi Bethune Hospital, Shanxi Academy of Medical Sciences, Taiyuan, 030002 China

**Keywords:** Epidemiology, Prevalence, Visual impairment, Blindness, Refractive error, COVID-19, China, Health policy, Public health

## Abstract

This cross-sectional study aims to investigate the prevalence and causes of visual impairment (VI) and blindness in Jiangsu Province, China in 2022 during the COVID-19 pandemic. Participants (n = 13,208, aged 18–93) underwent comprehensive ocular examinations. The prevalence and causes of binocular VI (presenting visual acuity [VA] ≥ 20/400 and < 20/63 in the better eye) and blindness (presenting VA < 20/400 in the better eye) were assessed according to the World Health Organization (WHO) criteria. The estimation of refractive error prevalence was conducted using the following classification: myopia ≤  − 0.50 diopters (D), high myopia ≤  − 6.00 D, hyperopia ≥ 0.50 D, and anisometropia ≥ 1.00 D. The overall prevalence of binocular VI and blindness was 21.04% (95% confidence interval [CI] 20.35–21.74%) and 0.47% (95% CI 0.37–0.60%). The highest prevalence of binocular VI was in the population aged 18–24 years old (46.29%, [95% CI 44.30–48.28%]), those with education at university and above (43.47%, [95% CI 41.93–45.02%]), students (54.96%, [95% CI 52.73–57.17%]). Uncorrected refractive error (URE) was the leading cause of presenting binocular VI (93.40%) and blindness (50.79%). The prevalence of myopia was 54.75% (95% CI 53.90–55.60%). Actions are needed to control URE and myopia within the adult Chinese population, with a particular emphasis on the younger, well-educated demographic.

## Introduction

Coronavirus disease 2019 (COVID-19) was first reported in Wuhan, China, in mid-December 2019^1^. A novel coronavirus was declared by the World Health Organization (WHO) on March 11, 2020^1–3^. To stop its spread, the Chinese government implemented a series of aggressive policies, including school closures, home quarantines, and the lockdown of social entertainment activities^4,5^. These strategies have infected and changed residents’ lifestyles and eye health to varying degrees^3^. For example, timely treatment was unavailable, outdoor activities were reduced, and the use of electronic instruments was more frequent^3,6,7^. Previous studies have reported a rapid increase in visual impairment (VI) in Chinese students and a higher prevalence of VI in students with more screen-based activities during the COVID-19 pandemic^6,7^. The Chinese government decided to stop epidemic surveillance and open socialization in December, 2022^8^. Contemporary and accurate data on the prevalence of blindness and vision impairment in 2022 are critical and fundamental for public health policy formulation, scientific advances, and industry research implementation after the end of the epidemic.

Blindness and VI cause loss of productivity and quality of life, increase the social burden and cause serious public concern^9^. In recent decades, the Chinese government has proposed strategies to prevent and treat VI and blindness, such as improving the affordability of eye care and providing free clinical treatments^10^. In addition, the Chinese population has increased their attention to eye health and visual function. These changes reduced the prevalence of blindness in the Chinese population from 1990 to 2019 to slightly below the global average^11^. However, due to an increase in population age, obesity, and diabetes, the prevalence of VI in China increased from 1990 to 2019^11^. With the increased rate of cataract surgery^12^ and widespread use of anti-VEGF treatment for age-related macular degeneration (AMD)^13^ and diabetic retinopathy (DR)^14^, the major causes of VI and blindness in Chinese adults might have changed. To date, the cause-specific prevalence of blindness and VI among adults in China during the COVID-19 pandemic has not been reported.

Jiangsu Province is located in the Yangtze River Delta region on the eastern coast of mainland China. Jiangsu's regional development, People's Livelihood Index, and per capita gross domestic product (GDP) rank first among all the provinces in China^15^. This study aimed to investigate the age-, gender-, and cause-specific prevalence of blindness and distance VI, epidemiological characteristics, and factors influencing refractive error among adults in Jiangsu Province of China in 2022 during the COVID-19 pandemic.

## Methods

### Study design

This cross-sectional study was conducted from January 2022 to December 2022, during the COVID-19 pandemic in Jiangsu Province, China, using a multistage sampling method. The primary data were collected in Jiangsu Province; data access, analysis, and reporting were permitted by the "Eye Health Survey in Fifteen Provinces of China" project team. This survey was approved by the Ethics Committee of Beijing Tongren Hospital, Capital Medical University (Number TRECKY2021-135). The requirement for written informed consent was waived. Study information was provided through an informed consent form. Verbal informed consent from subjects or their legally authorized representatives was obtained and documented in the records as previously described^16^. This study was conducted in accordance with the tenets of the World Medical Association Declaration of Helsinki.

The survey was conducted using a multistage sampling method based on the natural population. The sample size was calculated using the previously described sampling estimation method^16,17^ with the following formula: N = $(\frac{{Z}_{1-\frac{\mathrm{\alpha }}{2}}}{\delta }$)^2^*p*(1-p)*deff. N = sample size;$\delta$ = permissible errors; α = inspection level; p = the actual rate of main outcome indicators; deff = design effect. In this study, α = 0.05, deff = 1.5, and expected response rate ≥ 85%. The sample size estimation was conducted using the previously published prevalence of blindness and myopia in Jiangsu Province^11,17^, respectively, taking the maximum values. Considering a certain non-response rate (the expected response rate was no less than 85%), the sample size of this study was determined to be 13,092. Based on the economic and traffic situations, epidemiological survey experience, and equipment conditions, a survey of Jiangsu Province was conducted in Lianyungang City. Based on administrative villages and the resident population, the sample was further divided into basic sampling units (BSUs) with 1000 individuals per unit (all ages). BSUs were numbered and sorted. Based on the age distribution of the population, 16 BSUs were randomly selected with equal probabilities. All households within the selected BSUs were visited to enumerate those aged ≥ 18 years old and verify long-term residence (length of residence > 6 months). After screening, a total of 13,929 residents aged 18 and above were included in the investigation, meeting the required sample size for this survey. Individuals meeting eligibility requirements were informed and invited to participate in this study by corresponding clerks or community workers before the investigation team was stationed. Examination stations were set up in the nearby community for participants’ convenience.

All participants underwent a comprehensive eye examination which included presenting visual acuity (presenting VA; wearing present correction if any), best-corrected visual acuity (BCVA; logMAR visual acuity), intraocular pressure (IOP; non-contact tonometer), anterior and posterior segment examination. A face-to-face questionnaire interview was also given to collect information including age, gender, income, educational level, marital status, occupation, medical history and family history. Further details of the study protocol and examination procedure are provided in the Supplementary Materials S1.

### Definitions of visual impairment and blindness

There are two major definitions for blindness and VI. First, blindness and VI can be defined based on presenting VA^18,19^. The second definition of blindness and VI is based on BCVA^20,21^. Both definitions were used in this study.

The WHO defines blindness as presenting VA worse than 20/400 in the better-seeing eye, and VI as presenting VA worse than 20/63 to 20/400 (inclusive) in the better-seeing eye according to the 11th edition of the International Classification of Diseases^22^. The United States (US) criterion for blindness is VA worse than 20/200 in the better-seeing eye and VI is VA worse than 20/40 to 20/200 (inclusive) in the better-seeing eye.

### Definitions of causes of blindness and vision impairment

URE (including aphakia) was defined as presenting visual acuity of 20/40 or less in the better-seeing eye but improved to 20/32 or better with correction in that eye. Cataract was defined according to the Lens Opacities Classification System III. The other causes include undetermined, unidentified or specified causes that did not fit into the above categories. The diagnosis of glaucoma was in accordance with the International Society of Geographic and Epidemiological Ophthalmology classification. AMD was determined according to the Wisconsin Age-related Maculopathy Grading System. The diagnoses of amblyopia, glaucoma, DR, ocular trauma, optic neuropathy, corneal opacity, uveitis, myopic retinopathy followed the clinical standards and previous literature. The detailed definitions of causes of blindness and vision impairment were provided in the Supplementary Materials S1.

### Definitions of hyperopia, myopia, emmetropia, and anisometropia

The spherical equivalent refraction (SER) was calculated as the sum of the sphere power and half-cylinder power. Hyperopia was defined as SER ≥ 0.50 D. Hyperopia was divided into three stages: mild hyperopia (SER ≥ 0.50 D, ≤ 3.00 D), moderate hyperopia (SER > 3.00 D, ≤ 5.00 D) and high hyperopia (SER > 5.00 D). Myopia was defined as SER ≤  − 0.50 D. Myopia was further divided into three stages: mild myopia (SER ≤  − 0.50 D, >  − 3.00 D), moderate myopia (SER ≤  − 3.00 D, >  − 6.00 D), and high myopia (SER ≤  − 6.00 D). Anisometropia was defined as a difference between the right and left SERs of 1.00 D or above. Emmetropia was defined as an SER >  − 0.50 D and < 0.50 D^23,24^.

### Data analysis

Continuous variables with a normal distribution are presented as mean, followed by standard deviation (SD). The abnormal distribution of continuous variables was introduced as median, standard deviation (SD), and interquartile range from the first to the third quartile (Q1–Q3). Student's t-test and the Wilcoxon rank-sum test were used for normally and abnormally distributed quantitative data, respectively. Chi-squared or Fisher's exact tests were used to analyze categorical variables. The prevalence of VI and blindness was calculated as the ratio of participants with VI or blindness to the total number of participants. Estimates are presented as 95% confidence intervals (95% CIs). χ^2^ tests were used to evaluate gender-specific and age-specific differences between groups. Multivariate logistic regression was used to assess factors associated with VI, blindness, myopia, and hyperopia. Results are expressed as adjusted odds ratios (ORs) with 95% CIs. *P* values < 0.05 were considered statistically significant. Statistical analyses were performed using Stata SE 13 (Serial number 401306302851), R software (version 4.2.0, http://cran.r-project.org/), easy-R (www.empowerstats.com), and Prism (https://www.graphpad.com/scientific-software/prism/). Figures were created using GraphPad software (version 9.2.0).

### Ethics approval and consent to participant

This survey was approved by Ethics Committee of Beijing Tongren Hospital, Capital Medical University (Number TRECKY2021-135), and the requirement for written informed consent was waived.

## Results

### Characteristics of the study group

A total of 13,929 eligible individuals were enumerated, 13,208 of whom completed the eye examination and face-to-face interview (response rate 94.82%). Among 13,208 individuals examined, 56.86% were female. The mean age of participants was 47.02 ± 18.59 (SD) years (range, 18–93 years). The mean body mass index was 23.67 ± 3.27 (SD). Of all the ethnic groups, 99.27% of participants were self-reported Han people. The proportion of participants with high (> 12 years), medium (> 9–12 years) and low (≤ 9 years) educational levels were 43.72%, 18.21% and 38.07%, respectively. The proportion of working participants, unworking participants, and students were 55.83%, 29.60%s and 14.57%, respectively. 72.03% of participants had yearly personal income ≥ 10,000 RMB. 78.88% of participants were married. The detailed distribution of participants by age, nationality, educational level, occupational status, income level and marital status are shown in Supplementary Materials S1 and Supplementary Table S1.

Eye examination results showed that 337 (2.55%) participants had cataracts. A total of 269 (2.04%) participants had cataract surgeries, including 87 participants who had cataract surgeries for both eyes and 8 participants who had no intraocular lens implantation for one eye after cataract surgery. The mean IOP was 13.83 ± 2.46 mmHg (range from 6.00 to 47.00 mmHg). Besides, 2762 (20.91%) participants wore glasses daily (Supplementary Table S2) and 169 (1.28%) participants had refractive surgery (Supplementary Table S3). More details about the study group characteristics are presented in the Supplemental Material S1 and Supplementary Tables S1–S4.

### Age- and gender-specific prevalence of visual impairment and blindness

**Figure 1:**
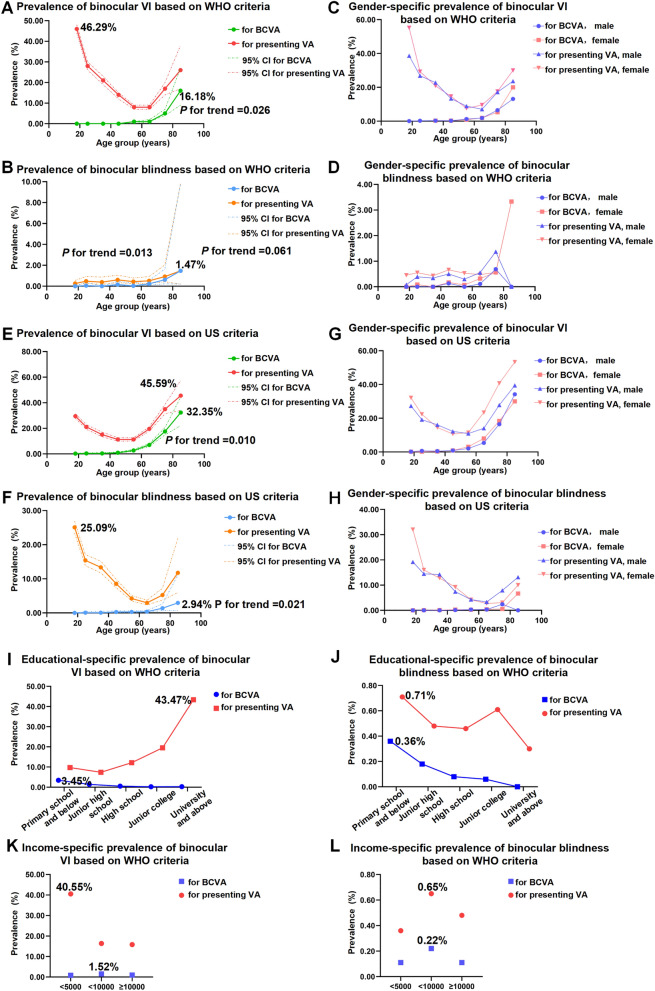
Age-, gender- educational- and income-specific prevalence of visual impairment (VI) and blindness. (**a**–**d**) The prevalence of VI and blindness based on WHO criteria. (**e–h**) The prevalence of VI and blindness based on US criteria. (**i**,**j**) The educational-specific prevalence of VI and blindness based on WHO criteria. (**k**,**l**) The income-specific prevalence of VI and blindness based on WHO criteria. The highest prevalence and* P* value for trend was marked in the figure.

Using the WHO criterion, the overall prevalence of binocular VI was 0.99% (95% CI 0.84–1.18%) for BCVA and 21.04% (95% CI 20.35–21.74%) for presenting VA. The overall prevalence of binocular blindness was 0.11% (95% CI 0.07–0.19%) for BCVA and 0.47% (95% CI 0.37–0.60%) for presenting VA (Table 1). The prevalence of binocular VI and blindness for BCVA and the prevalence of blindness for presenting VA were higher in successively older individuals (*P* for trend = 0.026, 0.061, and 0.013, respectively), as shown in Fig. 1a,b and Table 1. However, the prevalence of binocular presenting VI gradually decreased with age in individuals aged 18–64 years and gradually increased with age in individuals aged ≥ 65 years (Fig. 1a and Table 1). The highest prevalence of binocular VI was observed in individuals aged 18–24 years old (46.29% [95% CI 44.30–48.28%]). No significant gender-specific differences were observed, as shown in Fig. 1c,d (χ^2^ = 4.89, *P* = 0.674 for VI and χ^2^ = 2.39, *P* = 0.794 for blindness based on BCVA; χ^2^ = 13.89, *P* = 0.053 for VI and χ^2^ = 4.88, *P* = 0.674 for blindness based on presenting VA).

**Table 1:** Prevalence of binocular visual impairment and blindness for presenting visual acuity and BCVA (Overall and by age and gender, using the WHO criteria).

Group	Age (years)	No. of participants	Visual impairment				Blindness			
			Best corrected visual acuity		Presenting visual acuity		Best corrected visual acuity		Presenting visual acuity	
			No	% (95% CI)	No	% (95% CI)	No	% (95% CI)	No	% (95% CI)
Men	18–24	1303	1	0.08 (0.01–0.54)	504	38.68 (36.07–41.36)	0	-	1	0.08 (0.01–0.54)
	25–34	766	2	0.26 (0.07–1.04)	206	26.89 (23.87–30.15)	0	-	3	0.39 (0.13–1.21)
	35–44	596	2	0.34 (0.08–1.33)	136	22.82 (19.62–26.36)	0	-	2	0.34 (0.08–1.33)
	45–54	794	2	0.25 (0.06–1.00)	106	13.35 (11.16–15.90)	1	0.13 (0.02–0.89)	4	0.50 (0.19–1.34)
	55–64	1006	13	1.29 (0.75–2.21)	90	8.95 (7.33–10.88)	0	-	3	0.30 (0.10–0.92)
	65–74	904	17	1.88 (1.17–3.00)	64	7.08 (5.58–8.95)	1	0.11 (0.02–0.78)	5	0.55 (0.23–1.32)
	75–84	291	19	6.53 (4.20–10.02)	50	17.18 ()13.26–21.97)	2	0.69 (0.17–2.71)	4	1.37 (0.52–3.61)
	≥ 85	38	5	13.16 (5.52–28.22)	9	23.68 (12.70–39.84)	0	-	0	-
	Total	5698	61	1.07 (0.83–1.37)	1165	20.45 (19.42–21.51)	4	0.07 (0.03–0.19)	22	0.39 (0.25–0.59)
Women	18–24	1108	0	-	612	55.23 (52.29–58.14)	0	-	5	0.45 (0.19–1.08)
	25–34	1091	0	-	320	29.33 (26.70–32.10)	1	0.09 (0.01–0.65)	6	0.55 (0.25–1.22)
	35–44	938	3	0.32 (0.10–0.99)	196	20.90 (18.41–23.62)	0	-	4	0.43 (0.16–1.13)
	45–54	1211	4	0.33 (0.12–0.88)	178	14.70 (12.81–16.81)	2	0.17 (0.04–0.66)	8	0.66 (0.33–1.32)
	55–64	1518	13	0.86 (0.50–1.47)	116	7.64 (6.41–9.09)	1	0.07 (0.01–0.47)	8	0.53 (0.26–1.05)
	65–74	1256	25	1.99 (1.35–2.93)	120	9.55 (8.05–11.31)	4	0.32 (0.12–0.85)	6	0.48 (0.21–1.06)
	75–84	358	19	5.31 (3.41–8.18)	63	17.60 (13.99–21.90)	2	0.56 (0.14–2.21)	2	0.56 (0.14–2.21)
	≥ 85	30	6	20.00 (9.14–38.31)	9	30.00 (16.22–48.68)	1	3.33 (0.45–20.76)	1	3.33 (0.45–20.76)
	Total	7510	70	0.93 (0.74–1.18)	1614	21.49 (20.58–22.44)	11	0.15 (0.08–0.26)	40	0.53 (0.39–0.73)
Men and women	18–24	2411	1	0.04 (0.01–0.29)	1116	46.29 (44.30–48.28)	0	-	6	0.25 (0.11–0.55)
	25–34	1857	2	0.11 (0.03–0.43)	526	28.33 (26.32–30.42)	1	0.05 (0.01–0.38)	9	0.48 (0.25–0.93)
	35–44	1534	5	0.33 (0.14–0.78)	332	21.64 (19.65–23.78)	0	-	6	0.39 (0.18–0.87)
	45–54	2005	6	0.30 (0.13–0.66)	284	14.16 (12.71–15.76)	3	0.15 (0.05–0.46)	12	0.60 (0.34–1.05)
	55–64	2524	26	1.03 (0.70–1.51)	206	8.16 (7.15–9.30)	1	0.04 (0.01–0.28)	11	0.44 (0.24–0.79)
	65–74	2160	42	1.94 (1.44–2.62)	184	8.52 (7.41–9.77)	5	0.23 (0.10–0.56)	11	0.51 (0.28–0.92)
	75–84	649	38	5.86 (4.29–7.95)	113	17.41 (14.68–20.53)	4	0.62 (0.23–1.63)	6	0.92 (0.42–2.04)
	≥ 85	68	11	16.18 (9.15–27.00)	18	26.47 (17.30–38.25)	1	1.47 (0.20–9.84)	1	1.47 (0.20–9.84)
	Total	13,208	131	0.99 (0.84–1.18)	2779	21.04 (20.35–21.74)	15	0.11 (0.07–0.19)	62	0.47 (0.37–0.60)
Standardized	≥ 45	7406	123	1.66 (1.39–1.98)	805	10.87 (10.18–11.60)	14	0.19 (0.11–0.32)	41	0.55 (0.41–0.75)
	≥ 55	5401	117	2.17 (1.81–2.59)	521	9.65 (8.89–10.46)	11	0.20 (0.11–0.37)	29	0.54 (0.37–0.77)
	≥ 65	2877	91	3.16 (2.58–3.87)	315	10.95 (9.86–12.14)	10	0.35 (0.19–0.64)	18	0.63 (0.39–0.99)

Using the US criterion, the overall prevalence of binocular VI was 2.95% (95% CI 2.68–3.26%) for BCVA and 19.12% (95% CI 18.46–19.80%) for presenting VA using the WHO criterion. The overall prevalence of binocular blindness was 0.25% (95% CI 0.18–0.35%) for BCVA and 11.21% (95% CI 10.68–11.75%) for presenting VA (Supplementary Table S5). For BCVA, the prevalence of binocular VI and blindness was higher in older individuals (*P* for trend = 0.010 and 0.021, respectively), as shown in Fig. 1e,f and Supplementary Table S5. For presenting VA, the prevalence of binocular VI decreased with age in individuals aged 18–54 years and increased with age in individuals aged ≥ 55 years (Fig. 1e, Supplementary Table S5). The highest prevalence of binocular presenting VI was in individuals aged ≥ 85 years (45.59%, 95% CI 34.12–57.54%). The prevalence of binocular blindness decreased with age in individuals aged 18–74 years and increased in individuals aged ≥ 75 years (Fig. 1f, Supplementary Table S5). The highest prevalence of presenting blindness was observed in individuals aged 18–24 years (25.09%, 95% CI 23.40%–26.86%). No gender-specific significant difference was observed based on BCVA (χ^2^ = 12.93, *P* = 0.074 for VI and χ^2^ = 8.66, *P* = 0.194 for blindness based on BCVA). Based on presenting VA, the gender-specific difference was significant (χ^2^ = 50.56, *P* < 0.001 for VI and χ^2^ = 16.05, *P* = 0.025 for blindness based on presenting VA), as shown in Fig. 1g,h.

### Education-, marriage-, income-, and occupation-specific prevalence of visual impairment and blindness

**Figure 2:**
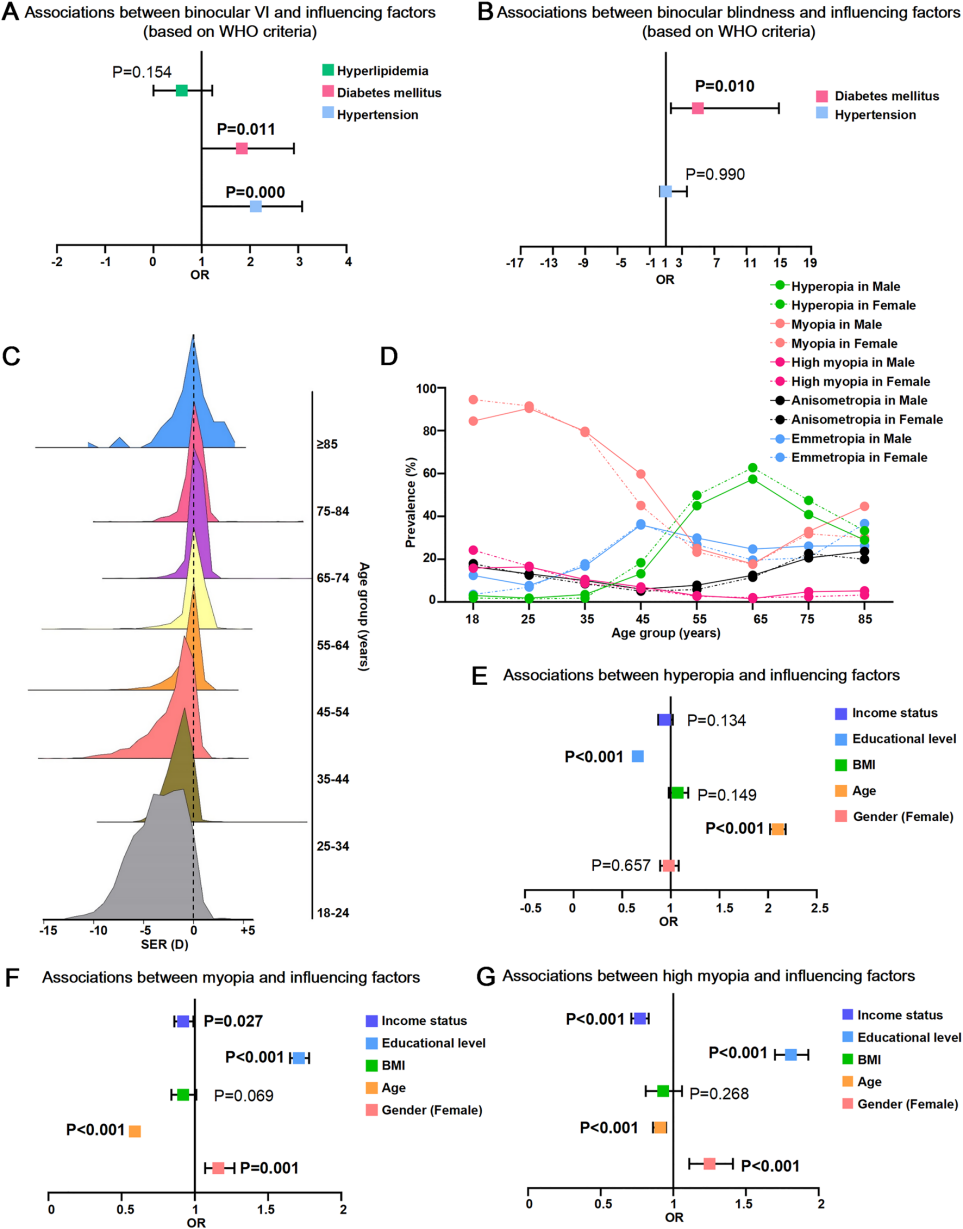
Associations between influencing factors and the prevalence of binocular visual impairment (VI), binocular blindness, hyperopia, myopia and high myopia. (**a**,**b**) Forest image showing associations between the prevalence of binocular best-corrected VI, blindness and hyperlipidemia, diabetes mellitus, hypertension using multivariate logistic regression analysis. (**c**) The mountain maps showing distribution of refractive error in different age groups. (**d**) Age- and gender-specific prevalence of hyperopia, myopia, high myopia, anisometropia and emmetropia. (**e**–**g**) The forest images showing associations between the prevalence of hyperopia, myopia, high myopia and income level, educational level, age, gender, body mass index (BMI) using multivariate logistic regression analysis.

Using WHO standards, we analyzed the prevalence of binocular VI and blindness according to educational, marital, income, and occupational status. Our results showed that the prevalence of binocular VI decreased with the educational level for BCVA but increased with the educational level for presenting VA (Fig. 1i). The highest prevalence of binocular VI for presenting VA was 43.47% (95% CI 41.93–45.02%) in individuals with university education and above. The highest prevalence of binocular VI for BCVA was 3.45% (95% CI 2.68–4.44%) in individuals with primary school education and below (Supplementary Table S6). The prevalence of binocular blindness generally decreased with the educational level for BCVA and VA (Fig. 2j). The highest prevalence of binocular blindness was 0.36% (95% CI 0.16–0.79%) for BCVA and 0.71% (95% CI 0.41–1.25%) for presenting VA in individuals with primary school education and below (Supplementary Table S6).

Single participants had a higher prevalence of binocular VI for presenting VA, a lower prevalence of binocular VI for BCVA, and a lower prevalence of binocular blindness for both BCVA and presenting VA than married participants. Divorced participants had the highest prevalence of binocular blindness for presenting VA (Supplementary Table S6).

Based on income status, individuals with an annual income below 5000 RMB showed the highest prevalence of binocular VI for presenting VA (40.55% [95% CI 38.73–42.39%]). Individuals with an annual income between 5000 and 10,000 RMB showed the highest prevalence of binocular VI for BCVA and the highest prevalence of binocular blindness for BCVA and VA (Fig. 1k,l, Supplementary Table S6).

Based on occupation, students showed the highest prevalence of binocular VI for presenting VA (54.96% [95% CI 52.73–57.17%]). Civil servants showed the highest prevalence of binocular blindness for presenting VA (0.66% [95% CI 0.32–1.38%]). Retired individuals and those with missing occupations had the highest prevalence of binocular VI and blindness for BCVA (Supplementary Table S6).

### Causes of visual impairment and blindness

Based on the BCVA, the leading cause of binocular VI was cataracts (17.14%), followed by amblyopia, glaucoma, DR, ocular trauma, optic neuropathy, AMD, and corneal opacity (Table 2, Supplementary Fig. S1a). The leading causes of binocular blindness were cataracts (20.00%) and amblyopia (20.00%), followed by DR and uveitis (Table 2; Supplementary Fig. S1b). Similarly, the most common cause of monocular VI was cataracts (15.07%; Table 3, Supplementary Fig. S1e). The most common causes of monocular blindness were cataracts (15.38%) and amblyopia (15.38%; Table 3 and Supplementary Fig. S1f).

**Table 2:** Causes of binocular visual impairment and blindness based on BCVA and presenting visual acuity using the WHO criteria.

Causes	Number (%) of participants					
	Based on BCVA			Based on presenting visual acuity		
	VI	Blindness	Combined	VI	Blindness	Combined
Uncorrected refractive error (including aphakia)	–	–	–	2718 (93.40%)	32 (50.79%)	2750 (92.50%)
Cataract	24 (17.14%)	3 (20.00%)	27 (17.42%)	38 (1.31%)	8 (12.70%)	46 (1.55%)
Amblyopia	12 (8.57%)	3 (20.00%)	15 (9.68%)	14 (0.48%)	3 (4.76%)	17 (0.57%)
Glaucoma	5 (3.57%)	0 (0.00%)	5 (3.23%)	7 (0.24%)	0 (0.00%)	7 (0.24%)
Diabetic retinopathy	5 (3.57%)	2 (13.33%)	7 (4.52%)	6 (0.21%)	2 (3.17%)	8 (0.27%)
Ocular trauma	4 (2.86%)	0 (0.00%)	4 (2.58%)	5 (0.17%)	1 (1.59%)	6 (0.20%)
Age-related macular degeneration	2 (1.43%)	0 (0.00%)	2 (1.29%)	3 (0.10%)	0 (0.00%)	3 (0.10%)
Optic neuropathy	3 (2.14%)	0 (0.00%)	3 (1.94%)	3 (0.10%)	0 (0.00%)	3 (0.10%)
Corneal opacity	1 (0.71%)	0 (0.00%)	1 (0.65%)	1 (0.03%)	0 (0.00%)	1 (0.03%)
Uveitis	0 (0.00%)	1 (6.67%)	1 (0.65%)	0 (0.00%)	1 (1.59%)	1 (0.03%)
Myopic retinopathy	6 (4.29%)	0 (0.00%)	6 (3.87%)	0 (0.00%)	0 (0.00%)	0 (0.00%)
Other	78 (55.71%)	6 (40.00%)	84 (54.19%)	115 (3.95%)	16 (25.40%)	131 (4.41%)
Total participants	140 (100%)	15 (100%)	155(100%)	2910 (100%)	63 (100%)	2973 (100%)

*BCVA* best-corrected visual acuity, *VI* visual impairment, *WHO* World Health Organization.

**Table 3:** Causes of monocular visual impairment and blindness based on BCVA and presenting visual acuity using the WHO criteria.

Causes	Number (%) of eyes					
	Based on BCVA			Based on presenting visual acuity		
	VI	Blindness	Combined	VI	Blindness	Combined
Uncorrected refractive error (including aphakia)	–	–	–	6156 (90.53%)	100 (29.07%)	6256 (87.57%)
Cataract	80 (15.07%)	30 (15.38%)	110 (15.15%)	75 (1.10%)	40 (11.63%)	115 (1.61%)
Amblyopia	30 (5.65%)	30 (15.38%)	60 (8.26%)	32 (0.47%)	31 (9.01%)	63 (0.88%)
Glaucoma	18 (3.39%)	6 (3.08%)	24 (3.31%)	19 (0.28%)	8 (2.33%)	27 (0.38%)
Diabetic retinopathy	4 (0.75%)	10 (5.13%)	14 (1.93%)	5 (0.07%)	10 (2.91%)	15 (0.21%)
Ocular trauma	24 (4.52%)	15 (7.69%)	39 (5.37%)	22 (0.32%)	20 (5.81%)	42 (0.59%)
Age-related macular degeneration	7 (1.32%)	4 (2.05%)	11 (1.52%)	8 (0.12%)	4 (1.16%)	12 (0.17%)
Optic neuropathy	10 (1.88%)	4 (2.05%)	14 (1.93%)	11 (0.16%)	5 (1.45%)	16 (0.22%)
Corneal opacity	4 (0.75%)	2 (1.03%)	6 (0.83%)	4 (0.06%)	2 (0.58%)	6 (0.08%)
Uveitis	0 (0.00%)	4 (2.05%)	4 (0.55%)	0 (0.00%)	4 (1.16%)	4 (0.06%)
Myopic retinopathy	11 (2.07%)	3 (1.54%)	14 (1.93%)	10 (0.15%)	4 (1.16%)	14 (0.20%)
Other	343 (64.60%)	87 (44.62%)	430 (59.23%)	458 (6.74%)	116 (33.72%)	588 (8.03%)
Total eyes	531 (100%)	195 (100%)	726 (100%)	6800 (100%)	344 (100%)	7144 (100%)

*BCVA* best-corrected visual acuity, *VI* visual impairment, *WHO* World Health Organization.

Based on presenting VA, the leading cause of binocular VI was uncorrected refractive error (93.40%, including aphakia and URE), followed by cataracts, amblyopia, glaucoma, DR, ocular trauma, AMD, optic neuropathy, and corneal opacity (Table 2, Supplementary Fig. S1c). The leading cause of binocular blindness was URE (50.79%), followed by cataracts, amblyopia, DR, ocular trauma, and uveitis (Table 2, Supplementary Fig. S1d). Consistent with previous results, the most common cause of monocular VI and blindness was also URE (90.53% and 29.07%, respectively; Table 3 and Supplementary Fig. S1g,h).

For participants of working age (aged 18–60 years for men and 18–55 years for women), the leading cause of binocular VI (98.92%) and blindness (69.44%) was URE, and the leading cause of monocular VI (98.07%) and blindness (50.37%) was URE based on the presenting VA. Based on the BCVA, cataracts were the leading cause of binocular and monocular VI (25.00% and 13.21% respectively). Based on the BCVA, the leading cause of binocular blindness was DR (25.00%), and the leading cause of monocular blindness was ocular trauma (13.51%; Supplementary Tables S7 and S8).

A multivariate logistic regression analysis was performed to explore factors associated with the prevalence of VI and blindness according to the WHO criteria. Our results showed that hypertension (OR = 2.12, 95% CI 1.45–3.08; *P* = 0.000) and diabetes mellitus (OR = 1.83, 95% CI 1.15–2.91; *P* = 0.011) were significantly associated with a higher prevalence of binocular VI based on BCVA (Supplementary Table S9 and Fig. 2a). Furthermore, diabetes mellitus (OR = 4.96, 95% CI 1.64–15.02; *P* = 0.010) was significantly associated with a higher prevalence of binocular blindness based on BCVA (Supplementary Table S9 and Fig. 2b).

### Prevalence of myopia, hyperopia, and anisometropia

To evaluate refractive error in the entire population, we did not exclude pseudophakic and aphakic patients or participants who had undergone refractive surgeries. In this study, 1.28% (169/13,208) participants underwent refractive surgeries, 2.04% (269/13,208) participants had cataract surgeries, and 1.98% (261/13,208) participants had intraocular lens implantation (Supplementary Table S3). Pearson’s correlation analysis revealed a significant correlation between the SERs of the right and left eyes (r = 0.8603, P < 0.001). The SERs of the right eye were used for further evaluation.

The mean SER of all eyes was − 1.49 ± 2.97 (SD) D, ranging from − 30.25 D to + 25.00 D. The most common refractive condition in the present study group was myopia (54.75% [95% CI 53.90–55.60%]), followed by hyperopia (24.94% [95% CI 24.21–25.68%]), emmetropia (20.31% [95% CI 19.63–21.00%]). The prevalence of anisometropia was 11.24% (95% CI 10.71–11.79%). The prevalence rates of mild, moderate, and high hyperopia were 23.92% (95% CI 23.20–24.66%), 0.79% (95% CI 0.66–0.96%), and 0.22% (95% CI 0.15–0.32%), respectively. The prevalence rates of mild, moderate, and high myopia were 29.23% (95% CI 28.46–30.01%), 16.46% (95% CI 15.84–17.10%), and 9.06% (95% CI 8.58–9.56%), respectively (Supplementary Table S10).

Figure 2c shows that the distribution of SER was asymmetric. In the younger age group, the highest number of participants was accompanied by higher myopic correction (Fig. 2c). Hyperopia was more common in participants aged ≥ 55 years. The most hyperopic age group was the 65–74-year-old group (60.51% [95% CI 58.43–62.55%]). The prevalence of myopia and high myopia increased with age from 18 to 74 years. A significant gender-specific difference was observed in the prevalence of myopia (χ^2^ = 7.60, *P* = 0.006). Myopia and high myopia were most prevalent in female participants aged 18–24 years (94.49% [95% CI 92.99-95.69%] for myopia and 24.28% [95% CI 21.84–26.89%] for high myopia). The most anisometropia age group was the ≥ 85-year-old group (Fig. 2d and Supplementary Table S11).

### Factors associated with hyperopia, myopia, and high myopia

Multivariate logistic regression analysis showed that older age (OR = 2.10, 95% CI 2.02–2.18; *P* < 0.001) and lower educational level (OR = 0.66, 95% CI 0.64–0.69; *P* < 0.001) were significantly associated with a higher prevalence of hyperopia (Supplementary Table S12, Fig. 2e). Furthermore, myopia was more prevalent in participants of the female (OR = 1.16, 95% CI 1.07–1.27; *P* = 0.001), younger age (OR = 0.59, 95% CI 0.57–0.61; *P* < 0.001), higher educational level (OR = 1.71, 95% CI 1.65–1.78; *P* < 0.001), and lower-income (OR = 0.92, 95% CI 0.86–0.99; *P* = 0.027), as shown in Supplementary Table S12 and Fig. 2f. Similarly, high myopia was more prevalent in participants of female (OR = 1.25, 95% CI 1.11–1.41; *P* < 0.001), younger age (OR = 0.91, 95% CI 0.86–0.95; *P* < 0.001), higher educational level (OR = 1.81, 95% CI 1.70–1.93; *P* < 0.001), and lower-income (OR = 0.77, 95% CI 0.71–0.83; *P* = 0.027) groups, as shown in Supplementary Table S12 and Fig. 2g.

## Discussion

This study clarified the prevalence and causes of distance VI and blindness in adults in Jiangsu Province, China, in 2022 during the COVID-19 pandemic. Our results showed that the prevalence of VI based on presenting VA in 2022 (21.04%) was much higher than the global prevalence in 1990 (3.83%), 2015 (2.95%), and 2022 (3.58%)^11,18,19,25^ as well as the previously reported prevalence in China and Jiangsu^11,19^ (Supplementary Table S13). Previous studies have indicated that the prevalence of binocular VI for presenting VA increases with age^11,25^. However, in the current study, the prevalence of binocular VI gradually decreased with age in individuals aged 18–64 years and gradually increased with age in individuals aged ≥ 65 years (Fig. 1a). Further analysis indicated that the population with the highest prevalence of binocular VI based on presenting VA was those who were aged 18–24 years (46.29%), with university education and above (43.47%), single status (47.33%), students (54.96%), and with an income below 5000 RMB per year (40.55%).

Similar to previous global and Chinese survey results, the most common reason for binocular VI was URE^11,18,19,25^. However, the proportion of URE-induced binocular VI in Jiangsu Province experienced a substantial increase, rising from 52.09% in 2019^11^ to 93.40% in 2022, and was strikingly higher compared to previous global data^11,18,19^ (Supplementary Table S13). After further investigation of the refractive error, we reported that the prevalence of myopia and high myopia in this study was 54.75% and 9.06%, respectively. The estimated prevalence of myopia and high myopia was 51.06% and 13.08%, respectively, in East Asia in 2020^26^ and 22.90% and 16.90%, respectively, in Beijing, China in 2001^27^ (Supplementary Table S14). These findings indicate that the notable rise in the prevalence of URE-induced binocular VI in Jiangsu Province can be attributed to the increasing prevalence of mild and moderate myopia rather than high myopia. Prevention, control strategies, and policies for high myopia are effective in China^28,29^.

We further compared the prevalence and causes of VI and blindness in individuals aged ≥ 50 years with previous studies^11,17–20,25,30,31^ (Supplementary Table S15). The prevalence of presenting VI and blindness in participants aged ≥ 50 years in Jiangsu Province in 2022 (10.13% and 0.58%, respectively) decreased substantially when compared with data from China in 2006 (31.70% and 2.29%, respectively) and 2014 (32.40% and 1.66%, respectively), and similar to the global prevalence in 2015 (10.41% and 1.90%, respectively)^11,17,19^. The major cause of presenting VI was URE, as previously reported^11,17,19,20,30^. However, the proportion of URE was greatly up-regulated (93.61% in 2022) compared to the global data (52.34% in 2015) and previous Chinese data (15.8% in 2014). The major cause of presenting blindness was cataracts, followed by URE, in global and Chinese data. However, in this study, the major cause of presenting blindness was URE (50.00%), followed by cataracts (Supplementary Table S15). In adults aged ≥ 50 years, the principal determinant of refractive error is the refractive lens power^32^. Previous studies have found that cortical cataracts can cause astigmatic shifts and nuclear cataracts can cause myopic shifts^27,33^. With the increase in cataract surgery rate in China^12^, more attention should be paid to correcting mild cataract-induced URE in individuals aged ≥ 50 years.

Based on BCVA, the prevalence of VI and blindness of Jiangsu participants aged ≥ 50 years in 2022 (1.88% and 0.20%, respectively) was slightly decreased when compared with the prevalence in China in 2004 (3.10% and 0.50%, respectively) and 2013 (5.10% and 1.00%, respectively)^21,30^. Similar to previous results, the major cause of BCVA-based VI and blindness was cataracts^20,21,30,31^. Nevertheless, the proportion of cataract-induced VI and blindness in 2022 (16.92% and 20.00%, respectively) underwent a substantial decrease when contrasted with the results in 2003 (71.80% and 44.70%, respectively) and 2013 (59.10% and 48.50%, respectively). The third cause of presenting VI and the second most common cause of best-corrected VI in China is macular degeneration^11,30^, which was replaced by amblyopia in 2022. The third most common cause of blindness, the second most common cause of best-corrected blindness in China, was glaucoma^11,30^, which was also replaced with amblyopia in 2022 (Supplementary Table S15). These results demonstrate the improved treatment of cataracts, AMD, and glaucoma in China in recent years.

For working-age participants, the leading cause of binocular and monocular best-corrected VI was cataracts, and the leading cause of binocular best-corrected blindness was DR. DR was the leading cause of blindness among working-age adults worldwide^34^. China and India are the two countries with the highest numbers of diabetes mellitus patients worldwide, with a rising prevalence of DR^35,36^. Ocular trauma is the leading cause of monocular best-corrected blindness in working-age individuals. Moreover, the proportion of ocular trauma-induced monocular blindness was higher in the male participants (21.74%) than in female participants (0.00%). Ocular trauma is a major cause of monocular BCVA-based visual impairment and blindness globally^37,38^. A survey of a rural population in northern China in 2006 reported that the proportion of trauma-induced monocular blindness was 21.0%, and men had a higher prevalence of ocular trauma compared to women^39^. Working-aged individuals assume heavy social and family responsibilities. Our results underscore the pressing need for eye care programs to prevent and treat DR and ocular trauma, particularly in the working-age Chinese population.

This study had some limitations. First, myopia was defined according to SER results, which were detected using non-cycloplegic autorefraction and small pupil optometry. The absence of cycloplegia may include accommodative spasms and an overestimation of myopic power. School closures, home quarantines, and social entertainment restrictions during the COVID-19 pandemic have increased excessive close work, such as long-time online courses and prolonged screen time, which might lead to accommodative spasms^40^. Mydriatic optometry using cycloplegic drops can effectively diagnose accommodative spasms. However, mydriatic optometry is inconvenient and time-consuming and can cause side effects in large-population surveys. A previous study showed that a combination of uncorrected visual acuity and non-cycloplegic autorefraction produced the highest net benefits for myopia screening^41^. In the future, strategies are required to rapidly exclude the influence of accommodative spam. Second, other causes contributed 3.95% of presenting VI and 25.40% of presenting blindness in this study, which remains large. The proportion of other causes of presenting VI and blindness was below the values from global data in 2015 (13.16% and 25.46%, respectively)^19^ and the data from China in 2019 (10.34% and 40.36%, respectively)^11^. One reason for this reduction in causal proportion was that we included patients with myopic retinopathy and amblyopia in this study, whereas previous studies did not. Another reason may be that other causes are curable and preventable. Future studies should include more low-prevalence causes such as trachoma. Third, uncorrected visual acuity (without spectacle or contact lenses if worn) and unaided visual acuity (without refractive error correction if spectacle or contact lenses were not worn) were not measured in this study. WHO recommended reporting uncorrected visual acuity in epidemiological surveys, not only presenting VA, in the 11th revision of International Classification of Diseases to give effective coverage of refractive error correction^22^. In future studies, uncorrected and unaided visual acuity will be assessed. Fourth, the fundus examination is hampered by severe cataracts. Therefore, the prevalence outcomes of AMD, DR, optic neuropathy, and myopic retinopathy were analyzed.

## Conclusions

This study highlights a substantially increased burden of URE and myopia among residents aged ≥ 18 years in Jiangsu Province of China in 2022 during the COVID-19 pandemic. Public awareness campaigns and policies need to intensify their efforts to control URE and myopia, with a particular emphasis on well-educated students aged 18–24 years. Moreover, priority attention should be directed towards addressing URE in individuals aged ≥ 50 years, as well as implementing effective measures for the treatment and prevention of diabetic retinopathy and ocular trauma in working-age population.

### Supplementary Information

Supplementary Information.

## Data Availability

The raw data of this study are available from the corresponding author upon reasonable request.

## Abbreviations

COVID-19: Coronavirus disease 2019
WHO: World Health Organization
BCVA: Best-corrected visual acuity
VA: Visual acuity
MSVI: Moderate to severe visual impairment
VI: Visual impairment
D: Diopter
SER: Spherical equivalent refraction
URE: Uncorrected refractive error
AMD: Age-related macular degeneration
DR: Diabetic retinopathy
US: United States
BSU: Basic sampling unit
CI: Confidence intervals
OR: Odds ratios
SD: Standard deviation
IOP: Intraocular pressure
BMI: Body mass index
