# Supplementary material for: Prevalence and causes of blindness and distance visual impairment in Chinese adult population in 2022 during the COVID-19 pandemic: a cross-sectional study
Source: Sci Rep. 2024 Feb 16;14:3890. doi: 10.1038/s41598-024-54325-0 (PMC10873313; doi:10.1038/s41598-024-54325-0)
Supplement: Supplementary file 1 — Supplementary Information. [file 41598_2024_54325_MOESM1_ESM.docx]

**Supplementary Materials**

**Prevalence and Causes of Blindness and Distance Visual Impairment in Chinese Adult Population in 2022 during the COVID-19 Pandemic: A cross-sectional study.**

Hua Wang^#1^, Zhi Xu^#1^, Dandan Chen^1^, Huihui Li^1^, Junyan Zhang^*3^, Qinghuai Liu*^2^, Han Shen*^2^.

**Contents:**

1. Examination procedure.
2. Definitions of causes of blindness and vision impairment.
3. Characteristics of the study group.
4. Supplementary Table 1. Characteristics of the study group (N=13208).
5. Supplementary Table 2. Preliminary eye examination outcomes (N=13208).
6. Supplementary Table 3. Previous medical history (N=13208).
7. Supplementary Table 4. Family history (N=13208).
8. Supplementary Table 5. Prevalence of binocular visual impairment and blindness for presenting visual acuity and BCVA (Overall and by age and gender, using the US criteria).
9. Supplementary Table 6. Education-, Marriage-, Income- and occupation-specific prevalence of bilateral visual impairment using the WHO criteria.
10. Supplementary Table 7. Causes of binocular visual impairment and blindness at working age (using the WHO criteria).
11. Supplementary Table 8. Causes of monocular visual impairment and blindness at working age (using the WHO criteria).
12. Supplementary Table 9. Associations between risk factors and the prevalence of binocular visual impairment by multiple logistic regression analysis (using the WHO criteria).
13. Supplementary Table 10. Prevalence of hyperopia, myopia and anisometropia (N=13208).
14. Supplementary Table 11. Age-specific and gender-specific prevalence of hyperopia, myopia, emmetropia and anisometropia.
15. Supplementary Table 12. Factors associated with the prevalence of hyperopia, myopia and high myopia by multiple logistic regression analysis.
16. Supplementary Table 13. Comparison of the prevalence and causes of binocular visual impairment and blindness based on presenting visual acuity in population with all ages (Using the WHO criteria).
17. Supplementary Table 14. Comparison of the distribution of spherical equivalent refraction in population with all ages .
18. Supplementary Table 15. Comparison of the binocular visual impairment and blindness causes in Chinese and global populations aged ≥50 years old (Using the WHO criteria).
19. Supplementary Figure 1. Pie charts depicting the distribution of visual impairment (VI) and blindness causes using the WHO criteria.
20. References.

**Examination procedure**

All participants underwent ocular examinations in the following order: non-cycloplegic refraction test, presenting visual acuity (presenting VA; wearing present correction if any), best-corrected visual acuity (BCVA; logMAR visual acuity), intraocular pressure (IOP; non-contact tonometer), anterior segment examination, posterior segment examination, non-mydriatic color fundus photography. Sphere and cylinder refractive errors were detected by non-cycloplegic autorefractions with autorefractors (NIDEK AR-510A), followed by subjective refraction. BCVA was determined by subjective refraction without cycloplegia. A retroilluminated logarithm E chart at a distance of 4 m was used to conduct visual test, as previously described ^1-3^.

Anterior and posterior segments examinations were conducted by an experienced ophthalmologist (D.C.) using a slit-lamp biomicroscopy and a +90-diopter lens or directed ophthalmoscopy before and after dilation of the pupil. The pupils of patients with a BCVA of 20/40 or worse were dilated. Individuals at high risk of angle-closure glaucoma underwent eye examinations in a small-pupil situation. The principle cause of VI and blindness was determined by one examining ophthalmologist (D.C.) with an itemized list including uncorrected refractive error (including aphakia, URE), cataract, amblyopia, glaucoma, diabetic retinopathy (DR), ocular trauma, age-related macular degeneration (AMD), optic neuropathy, corneal opacity, uveitis, myopic retinopathy and other cause (including undetermined cause). If there was uncertainty regarding the primary causes of visual impairment (VI) and blindness, a consensus was reached through discussions with two senior ophthalmologists (H.W. and Z.X.) based on ocular records. When multiple causes involved, the examiner generally favored the most treatable or preventable cause based on World Health Organization (WHO) recommendations.

Information about age, gender, height, body weight, present and past medical history, family history, educational and marital status, occupation, personal financial situation was collected before eye examinations by face-to face questionnaire interviews.

All data were recorded on the examination days using an online application and subsequently underwent self-inspection (D.C.) and auditor inspection (H.L.). All staff members of this survey underwent trainings about the project, eye examination, data management and quality control. The National Blindness Prevention Guidance Team conducted a reexamination of the uploaded data.

**Definitions of causes of blindness and vision impairment**

URE (including aphakia) was defined as participants with or without existing correction who had a presenting visual acuity of 20/40 or less in the better seeing eye, but improved to 20/32 or better with correction in that eye ^1,4,5^. Cataract was defined as lens opacity that obscured observation of the fundus in accordance with the Lens Opacities Classification System III ^4,6^. Cataract was determined to be the principle cause of VI or blindness when the lens opacity was commensurate with VI or blindness. Amblyopia was defined as BCAV was 20/32 or worse with no underlying structural abnormality of the eye or visual pathway^2,7^. Glaucoma was diagnosed as subjects who had a vertical cup:disc ratio (VCDR) ≥0.85 or VCDR asymmetry ≥0.45 according to the International Society of Geographic and Epidemiological Ophthalmology classification ^8,9^. When optic dis cannot be seen, glaucoma was diagnosed as BCVA <20/400 and intraocular pressure ≥23.9mmHg or BCVA<20/400 with evidence of glaucoma filtering surgery or medical records available confirming glaucomatous visual morbidity ^8,9^. Visual field examinations were only conducted in suspicious glaucoma patients and patients with self-reported visual field loss after referral to the local hospital. Although the proportion of glaucoma as a cause of VI and blindness was likely to be underestimated without visual field test, this definition method has been used in previous epidemiological studies ^1,4,9^. DR was determined if the presence of intra-retinal neovascularization and/or microvascular abnormalities, and microaneurysms, hard exudates, hemorrhages, macular edema, cotton-wool spots according to the Chinese multidisciplinary expert consensus on the prevention and treatment of diabetic eye disease (2021 edition) ^10,11^. Ocular trauma was defined as self-reported history of any eye injury requiring medical attention with or without hospitalisation ^12-14^. AMD was diagnosed if exudative degeneration or geographic atrophy was observed on the macula according to the Wisconsin Age-related Maculopathy Grading System ^15,16^. Myopic retinopathy was determined if the spherical equivalent was greater than -6.0 diopters (D), axial length ≥26mm with typical degenerative myopic fundus changes ^2,4^. The diagnoses of optic neuropathy, corneal opacity and uveitis followed the clinical standards. The other causes include undetermined, unidentified or specified causes that did not fit into above causes ^17^.

**Characteristics of the study group**

In this study, 5775 (43.72%) subjects had high education level (>12 years, junior college and above), 5028 (38.07%) subjects had low education level (≤9 years, junior high school and below) and 2405 (18.21%) subjects had medium education level (>9-12 years, high school). A total of 7374 (55.83%) participants were at work. The most common occupation was enterprise staffs (22.49%), followed by self-employed people (17.64%), civil servants (8.00%) and property management personnel (7.70%). There were 3909 (29.60%) participants are retired or missed occupation, and 1925 (14.57%) participants were students. The estimated yearly personal income of most (72.03%) subjects was 10000 RMB or more. Only 20.99% subjects had estimated yearly personal income below 5000 RMB, and 6.98% subjects had estimated yearly personal income between 5000 and 10000 RMB. Most (78.88%) participants were married, and 20.99% participants were single (Supplementary Table 1).

Eye examination results showed that 337 (2.55%) participants had cataract, 34 (0.26%) participants had corneal opacity, 22 (0.17%) participants had glaucoma, 15 (0.11%) participants had DR, and 5 (0.04%) participants had AMD (Supplementary Table 2). The mean IOP was 13.83 ± 2.46 mmHg (range from 6.00 mmHg to 47.00 mmHg). A total of 269 (2.04%) participants had cataract surgeries, including which 87 participants had cataract surgeries for both eyes and 8 participants had no intraocular lens implantation for one eye after cataract surgery. Besides, 169 (1.28%) participants had refractive surgery (Supplementary Table 3).

A total of 2064 (15.63%) participants had hypertension, 924 (7.00%) participants had diabetes mellitus, 649 (4.91%) participants had hyperlipidemia, 45 (0.34%) participants had coronary heart disease and 17 (0.13%) participants had a history of stroke (Supplementary Table 3).There was no participant reported a family history of DR. Only 3 (0.02%) participants had family histories of glaucoma and 1 (0.01%) participant had a family history of macular degeneration (Supplementary Table 4).

**Supplementary Table 1.** Characteristics of the study group (N=13208).

| **Baseline characteristics** | Number of participants (proportion %) |
| --- | --- |
| **Gender** | |
| Male | 5698 (43.14%) |
| Female | 7510 (56.86%) |
| **Age (years)** | |
| 18-24 | 2411 (18.25%) |
| 25-34 | 1857 (14.06%) |
| 35-44 | 1534 (11.61%) |
| 45-54 | 2005 (15.18%) |
| 55-64 | 2524 (19.11%) |
| 65-74 | 2160 (16.35%) |
| 75-84 | 649 (4.91%) |
| ≥85 | 68 (0.51%) |
| **Nationality** | |
| Han | 13112 (99.27%) |
| Hui | 43 (0.33%) |
| Mongolia | 5 (0.04%) |
| Uyghur | 4 (0.03%) |
| Zhuang | 2 (0.02%) |
| Other | 42 (0.32%) |
| **Educational level** | |
| Primary school and below | 1679 (12.71%) |
| Junior high school | 3349 (25.36%) |
| High school | 2405 (18.21%) |
| Junior college | 1809 (13.70%) |
| University and above | 3966 (30.03%) |
| **Occupational status** | |
| Civil servants | 1057 (8.00%) |
| Enterprise staffs | 2970 (22.49%) |
| Self-employed people | 2330 (17.64%) |
| Property management personnel | 1017 (7.70%) |
| Retired or missing occupation | 3909 (29.60%) |
| Students | 1925 (14.57%) |
| **Income level** | |
| <5,000 RMB per year | 2772 (20.99%) |
| ≥5,000 and <10,000 RMB per year | 922 (6.98%) |
| ≥10,000 RMB per year | 9514 (72.03%) |
| **Marital status** | |
| Single | 2772 (20.99%) |
| Married | 10418 (78.88%) |
| Divorced | 15 (0.11%) |
| Widowed | 3 (0.02%) |

**Supplementary Table 2.** Preliminary eye examination outcomes (N=13208).

| **Preliminary eye examination outcomes** | Number of participants (proportion%) |
| --- | --- |
| **Binocular blindness or low vision based on presenting** **visual acuity (WHO criteria)** | |
| Blindness | 62 (0.47%) |
| Visual impairment | 2779 (21.04%) |
| Normal | 10367 (78.49%) |
| **Binocular blindness or low vision based on presenting visual acuity (US criteria)** | |
| Blindness | 1480 (11.21%) |
| Visual impairment | 2525 (19.12%) |
| Normal | 9203 (69.68%) |
| **Binocular blindness or low vision based on best-correlated visual acuity (WHO criteria)** | |
| Blindness | 15 (0.11%) |
| Visual impairment | 131 (0.99%) |
| Normal | 13062 (98.89%) |
| **Binocular blindness or low vision based on best-correlated visual acuity (US criteria)** | |
| Blindness | 33 (0.25%) |
| Visual impairment | 390 (2.95%) |
| Normal | 12785 (96.80%) |
| **Wearing glasses** | |
| No | 10446 (79.09%) |
| Yes | 2762 (20.91%) |
| **Cataract** | |
| No | 12804 (96.94%) |
| Yes | 337 (2.55%) |
| Unclear | 67 (0.51%) |
| **Glaucoma** | |
| No | 13167 (99.69%) |
| Yes | 22 (0.17%) |
| Unclear | 19 (0.14%) |
| **Diabetic retinopathy** | |
| No | 12284 (93.01%) |
| Yes | 15 (0.11%) |
| Unclear | 909 (6.88%) |
| **Macular degeneration** | |
| No | 13188 (99.85%) |
| Yes | 5 (0.04%) |
| Unclear | 15 (0.11%) |
| **Corneal opacity** | |
| No | 13174 (99.74%) |
| Yes | 34 (0.26%) |
| Unclear | 0 (0.00%) |

**Supplementary Table 3.** Previous medical history (N=13208).

| **Previous medical history** | Number of participants (proportion%) |
| --- | --- |
| **Had refractive surgeries** |  |
| No | 13039 (98.72%) |
| Yes | 169 (1.28%) |
| **Had cataract surgery** |  |
| No | 12939 (97.96%) |
| Both eye surgeries | 87 (0.66%) |
| One eye surgery | 182 (1.38%) |
| **Had intraocular lens implantation** |  |
| No | 12947 (98.02%) |
| Yes | 261 (1.98%) |
| **Hypertension** |  |
| NO | 11130 (84.27%) |
| Yes | 2064 (15.63%) |
| Unclear | 14 (0.11%) |
| **Diabetes mellitus** |  |
| NO | 12,256 (92.79%) |
| Yes | 924 (7.00%) |
| Unclear | 28 (0.21%) |
| **Hyperlipidemia** |  |
| NO | 12527 (94.84%) |
| Yes | 649 (4.91%) |
| Unclear | 32 (0.24%) |
| **Coronary heart disease** |  |
| NO | 13159 (99.63%) |
| Yes | 45 (0.34%) |
| Unclear | 4 (0.03%) |
| **Stroke** |  |
| NO | 13190 (99.86%) |
| Yes | 17 (0.13%) |
| Unclear | 1 (0.01%) |

**Supplementary Table 4.** Family history (N=13208).

| **Family history** | Number of participants (proportion%) |
| --- | --- |
| **Family history of glaucoma** | |
| No | 13205 (99.98%) |
| Yes | 3 (0.02%) |
| **Family history of macular degeneration** | |
| No | 13207 (99.99%) |
| Yes | 1 (0.01%) |
| **Family history of diabetic retinopathy** | |
| No | 13208 (100.00%) |
| Yes | 0(0.00%) |

**Supplementary Table 5.** Prevalence of binocular visual impairment and blindness for presenting visual acuity and best-corrected visual acuity (Overall and by age and gender, using the US criteria).

|  |  |  | Visual impairment | | | | Blindness | | | |
| --- | --- | --- | --- | --- | --- | --- | --- | --- | --- | --- |
|  |  |  | Best-corrected Visual Acuity | | Presenting Visual Acuity | | Best-corrected Visual Acuity | | Presenting Visual Acuity | |
| Group | Age (yrs.) | No. of Participants | No. | % (95% CI) | No. | % (95% CI) | No. | % (95% CI) | No. | % (95% CI) |
| Men | 18-24 | 1303 | 3 | 0.23 (0.07-0.71) | 356 | 27.32 (24.97-29.81) | 0 | - | 250 | 19.19 (17.14-21.42) |
|  | 25-34 | 766 | 5 | 0.65 (0.27-1.56) | 147 | 19.19 (16.55-22.14) | 0 | - | 111 | 14.49 (12.17-17.17) |
|  | 35-44 | 596 | 3 | 0.50 (0.16-1.55) | 96 | 16.11 (13.37-19.28) | 0 | - | 85 | 14.26 (11.68-17.31) |
|  | 45-54 | 794 | 7 | 0.88 (0.42-1.84) | 97 | 12.22 (10.11-14.69) | 1 | 0.13 (0.02-0.89) | 59 | 7.43 (5.80-9.48) |
|  | 55-64 | 1006 | 21 | 2.09 (1.36-3.18) | 110 | 10.93 (9.15-13.02) | 3 | 0.30 (0.10-0.92) | 43 | 4.27 (3.18-5.72) |
|  | 65-74 | 904 | 49 | 5.42 (4.12-7.10) | 127 | 14.05 (11.93-16.47) | 3 | 0.33 (0.11-1.02) | 30 | 3.32 (2.33-4.71) |
|  | 75-84 | 291 | 48 | 16.49 (12.65-21.22) | 81 | 27.84 (22.98-33.27) | 7 | 2.41 (1.15-4.97) | 23 | 7.90 (5.30-11.62) |
|  | ≥85 | 38 | 13 | 34.21 (20.86-50.63) | 15 | 39.47 (25.22-55.77) | 0 | - | 5 | 13.16 (5.52-28.22) |
|  | Total | 5698 | 149 | 2.61 (2.23-3.06) | 1029 | 18.06 (17.08-19.08) | 14 | 0.25 (0.15-0.41) | 606 | 10.64 (9.86-11.46) |
| Women | 18-24 | 1108 | 2 | 0.18 (0.05-0.72) | 356 | 32.13 (29.44-34.94) | 0 | - | 355 | 32.04 (29.36-34.85) |
|  | 25-34 | 1091 | 2 | 0.18 (0.05-0.73) | 244 | 22.36 (19.99-24.94) | 1 | 0.09 (0.01-0.65) | 175 | 16.04 (13.98-18.34) |
|  | 35-44 | 938 | 3 | 0.32 (0.10-0.99) | 136 | 14.50 (12.39-16.90) | 1 | 0.11 (0.02-0.75) | 120 | 12.79 (10.80-15.09) |
|  | 45-54 | 1211 | 11 | 0.91 (0.50-1.63) | 129 | 10.65 (9.03-12.52) | 4 | 0.33 (0.12-0.88) | 112 | 9.25 (7.74-11.02) |
|  | 55-64 | 1518 | 47 | 3.10 (2.33-4.10) | 175 | 11.53 (10.02-13.24) | 4 | 0.26 (0.10-0.70) | 64 | 4.22 (3.31-5.35) |
|  | 65-74 | 1256 | 101 | 8.04 (6.66-9.68) | 294 | 23.41 (21.15-25.83) | 5 | 0.40 (0.17-0.95) | 34 | 2.71 (1.94-3.77) |
|  | 75-84 | 358 | 66 | 18.44 (14.75-22.80) | 146 | 40.78 (35.80-45.96) | 2 | 0.56 (0.14-2.21) | 11 | 3.07 (1.71-5.47) |
|  | ≥85 | 30 | 9 | 30.00 (16.22-48.68) | 16 | 53.33 (35.52-70.33) | 2 | 6.67 (1.63-23.51) | 3 | 10.00 (3.20-27.21) |
|  | Total | 7510 | 241 | 3.21 (2.83-3.63) | 1496 | 19.92 (19.03-20.84) | 19 | 0.25 (0.16-0.40) | 874 | 11.64 (10.93-12.38) |
| Men & Women | 18-24 | 2411 | 5 | 0.21 (0.09-0.50) | 712 | 29.53 (27.74-31.38) | 0 | - | 605 | 25.09 (23.40-26.86) |
|  | 25-34 | 1857 | 7 | 0.38 (0.18-0.79) | 391 | 21.06 (19.26-22.97) | 1 | 0.05 (0.01-0.38) | 286 | 15.40 (13.83-17.12) |
|  | 35-44 | 1534 | 6 | 0.39 (0.18-0.87) | 232 | 15.12 (13.42-17.01) | 1 | 0.07 (0.01-0.46) | 205 | 13.36 (11.75-15.16) |
|  | 45-54 | 2005 | 18 | 0.90 (0.57-1.42) | 226 | 11.27 (9.96-12.73) | 5 | 0.25 (0.10-0.60) | 171 | 8.53 (7.38-9.83) |
|  | 55-64 | 2524 | 68 | 2.69 (2.13-3.40) | 285 | 11.29 (10.11-12.59) | 7 | 0.28 (0.13-0.58) | 107 | 4.24 (3.52-5.10) |
|  | 65-74 | 2160 | 150 | 6.94 (5.95-8.10) | 421 | 19.49 (17.87-21.22) | 8 | 0.37 (0.19-0.74) | 64 | 2.96 (2.33-3.77) |
|  | 75-84 | 649 | 114 | 17.57 (14.82-20.69) | 227 | 34.98 (31.40-38.73) | 9 | 1.39 (0.72-2.64) | 34 | 5.24 (3.77-7.24) |
|  | ≥85 | 68 | 22 | 32.35 (22.28-44.38) | 31 | 45.59 (34.12-57.54) | 2 | 2.94 (0.73-11.11) | 8 | 11.76 (5.96-21.90) |
|  | Total | 13208 | 390 | 2.95 (2.68-3.26) | 2525 | 19.12 (18.46-19.80) | 33 | 0.25 (0.18-0.35) | 1480 | 11.21 (10.68-11.75) |
| Standardized | ≥45 | 7406 | 372 | 5.02 (4.55-5.54) | 1190 | 16.07 (15.25-16.92) | 31 | 0.42 (0.29-0.59) | 384 | 5.18 (4.70-5.71) |
|  | ≥55 | 5401 | 354 | 6.55 (5.92-7.25) | 964 | 17.85 (16.85-18.89) | 26 | 0.48 (0.33-0.71) | 213 | 3.94 (3.46-4.50) |
|  | ≥65 | 2877 | 286 | 9.94 (8.90-11.09) | 679 | 23.60 (22.08-25.19) | 19 | 0.66 (0.42-1.03) | 106 | 3.68 (3.05-4.44) |

Abbreviations: No, number; WHO, World Health Organization.

# **Supplementary Table 6.** Education-, marriage-, income- and occupation-specific prevalence of bilateral visual impairment using the WHO criteria.

|  |  |  | Visual impairment | | | | Blindness | | | |
| --- | --- | --- | --- | --- | --- | --- | --- | --- | --- | --- |
|  |  |  | Best-Corrected Visual Acuity | | Presenting Visual Acuity | | Best-Corrected Visual Acuity | | Presenting Visual Acuity | |
|  |  | No. of Participants | No. | % (95% CI) | No. | % (95% CI) | No. | % (95% CI) | No. | % (95% CI) |
| Educational level | Primary school and below | 1679 | 58 | 3.45(2.68-4.44) | 163 | 9.71(8.38-11.22) | 6 | 0.36(0.16-0.79) | 12 | 0.71(0.41-1.25) |
|  | Junior high school | 3349 | 47 | 1.40(1.06-1.86) | 247 | 7.38(6.54-8.31) | 6 | 0.18(0.08-0.40) | 16 | 0.48(0.29-0.78) |
|  | High school | 2405 | 12 | 0.50(0.28-0.88) | 292 | 12.14(10.89-13.51) | 2 | 0.08(0.02-0.33) | 11 | 0.46(0.25-0.82) |
|  | Junior college | 1809 | 4 | 0.22(0.08-0.59) | 353 | 19.51(17.75-21.41) | 1 | 0.06(0.01-0.39) | 11 | 0.61(0.34-1.09) |
|  | University and above | 3966 | 10 | 0.25(0.14-0.47) | 1724 | 43.47(41.93-45.02) | 0 | - | 12 | 0.30(0.17-0.53) |
| Marital Status | Single | 2772 | 1 | 0.04(0.01-0.26) | 1312 | 47.33(45.48-49.19) | 1 | 0.04(0.01-0.26) | 8 | 0.29(0.14-0.58) |
|  | Married | 10418 | 130 | 1.25(1.05-1.48) | 1462 | 14.03(13.38-14.71) | 14 | 0.13(0.08-0.23) | 53 | 0.51(0.39-0.67) |
|  | Divorced | 15 | 0 | - | 5 | 33.33(14.13-60.30) | 0 | - | 1 | 6.67(0.87-36.84) |
|  | Widowed | 3 | 0 | - | 0 | - | 0 | - | 0 | - |
| Income status | <5000 | 2772 | 24 | 0.87(0.58-1.29) | 1124 | 40.55(38.73-42.39) | 3 | 0.11(0.03-0.34) | 10 | 0.36(0.19-0.67) |
|  | <10000 | 922 | 14 | 1.52(0.90-2.55) | 151 | 16.38(14.13-18.91) | 2 | 0.22(0.05-0.86) | 6 | 0.65(0.29-1.44) |
|  | ≥10000 | 9514 | 93 | 0.98(0.80-1.20) | 1504 | 15.81(15.09-16.56) | 10 | 0.11(0.06-0.20) | 46 | 0.48(0.36-0.64) |
| Occupational status | Civil servants | 1057 | 4 | 0.38(0.14-1.00) | 364 | 34.44(31.63-37.36) | 0 | - | 7 | 0.66(0.32-1.38) |
|  | Enterprise staffs | 2970 | 11 | 0.37(0.21-0.67) | 582 | 19.60(18.21-21.06) | 2 | 0.07(0.02-0.27) | 14 | 0.47(0.28-0.79) |
|  | Self-employed people | 2330 | 21 | 0.90(0.59-1.38) | 297 | 12.75(11.45-14.16) | 1 | 0.04(0.01-0.30) | 9 | 0.39(0.20-0.74) |
|  | Property management personnel | 1017 | 16 | 1.57(0.97-2.55) | 109 | 10.72(8.96-12.77) | 1 | 0.10(0.01-0.70) | 4 | 0.39(0.15-1.04) |
|  | Retired or missing occupation | 3909 | 69 | 1.77(1.40-2.23) | 369 | 9.44(8.56-10.40) | 9 | 0.23(0.12-0.44) | 23 | 0.59(0.39-0.88) |
|  | Students | 1925 | 10 | 0.52(0.28-0.96) | 1058 | 54.96(52.73-57.17) | 2 | 0.10(0.03-0.41) | 5 | 0.26(0.11-0.62) |

Abbreviations: No, number; WHO, World Health Organization.

# **Supplementary Table 7.** Causes of binocular visual impairment and blindness at working age (using the WHO criteria).

| Causes | Number (%) of participants | | | | | | | |
| --- | --- | --- | --- | --- | --- | --- | --- | --- |
|  | Based on BCVA | | | | Based on presenting VA | | | |
|  | VI | | Blindness | | VI | | Blindness | |
|  | Men(18-60y) | Women(18-55y) | Men(18-60y) | Women(18-55y) | Men(18-60y) | Women(18-55y) | Men(18-60y) | Women(18-55y) |
| Uncorrected refractive error (including aphakia) | NA | NA | NA | NA | 1001 (98.33%) | 1308 (99.39%) | 9 (69.23%) | 16 (69.57%) |
| Cataract | 2 (15.38%) | 3 (42.86%) | 0 (0.00%) | 0 (0.00%) | 6 (0.59%) | 4 (0.30%) | 1 (7.69%) | 1 (4.35%) |
| Amblyopia | 1 (7.69%) | 0 (0.00%) | 0 (0.00%) | 0 (0.00%) | 1 (0.10%) | 0 (0.00%) | 0 (0.00%) | 0 (0.00%) |
| Glaucoma | 0 (0.00%) | 0 (0.00%) | 0 (0.00%) | 0 (0.00%) | 0 (0.00%) | 0 (0.00%) | 0 (0.00%) | 0 (0.00%) |
| Diabetic retinopathy | 0 (0.00%) | 0 (0.00%) | 0 (0.00%) | 1 (33.33%) | 0 (0.00%) | 0 (0.00%) | 0 (0.00%) | 1 (4.35%) |
| Ocular trauma | 2 (15.38%) | 0 (0.00%) | 0 (0.00%) | 0 (0.00%) | 2 (0.20%) | 0 (0.00%) | 0 (0.00%) | 0 (0.00%) |
| Age-related macular degeneration | 0 (0.00%) | 1 (14.29%) | 0 (0.00%) | 0 (0.00%) | 0 (0.00%) | 1 (0.08%) | 0 (0.00%) | 0 (0.00%) |
| Optic neuropathy | 1 (7.69%) | 0 (0.00%) | 0 (0.00%) | 0 (0.00%) | 1 (0.10%) | 0 (0.00%) | 0 (0.00%) | 0 (0.00%) |
| Corneal opacity | 0 (0.00%) | 0 (0.00%) | 0 (0.00%) | 0 (0.00%) | 0 (0.00%) | 0 (0.00%) | 0 (0.00%) | 0 (0.00%) |
| Uveitis | 0 (0.00%) | 0 (0.00%) | 0 (0.00%) | 0 (0.00%) | 0 (0.00%) | 0 (0.00%) | 0 (0.00%) | 0 (0.00%) |
| Myopic retinopathy | 0 (0.00%) | 0 (0.00%) | 0 (0.00%) | 0 (0.00%) | 0 (0.00%) | 0 (0.00%) | 0 (0.00%) | 0 (0.00%) |
| Other | 7 (53.85%) | 3 (42.86%) | 1 (100.00%) | 2 (66.67%) | 7 (0.69%) | 3 (0.23%) | 3 (23.08%) | 5 (21.74%) |
| Total participants | 13 (100%) | 7 (100%) | 1 (100%) | 3 (100%) | 1018 (100%) | 1316 (100%) | 13（100%） | 23（100%） |

Abbreviations: NA, not applicable; VI, visual impairment; WHO, World Health Organization.

#

# **Supplementary Table 8.** Causes of monocular visual impairment and blindness at working age (using the WHO criteria).

| Causes | Number (%) of eyes | | | |  | | | |
| --- | --- | --- | --- | --- | --- | --- | --- | --- |
|  | Based on BCVA | | | | Based on presenting VA | | | |
|  | VI | | Blindness | | VI | | Blindness | |
|  | Men(18-60y) | Women(18-55y) | Men(18-60y) | Women(18-55y) | Men(18-60y) | Women(18-55y) | Men(18-60y) | Women(18-55y) |
| Uncorrected refractive error (including aphakia) | NA | NA | NA | NA | 2188 (97.07%) | 2904 (98.84%) | 28 (42.42%) | 40 (57.97%) |
| Cataract | 8 (13.33%) | 6 (15.00%) | 1 (4.35%) | 2 (14.29%) | 10 (44.37%) | 3 (0.10%) | 3 (4.55%) | 6 (8.70%) |
| Amblyopia | 2 (3.33%) | 6 (15.00%) | 2 (8.70%) | 1 (7.14%) | 2 (0.09%) | 6 (0.20%) | 2 (3.03%) | 1 (1.45%) |
| Glaucoma | 1 (1.67%) | 0 (0.00%) | 0 (0.00%) | 0 (0.00%) | 1 (0.04%) | 0 (0.00%) | 0 (0.00%) | 0 (0.00%) |
| Diabetic retinopathy | 0 (0.00%) | 0 (0.00%) | 0 (0.00%) | 2 (14.29%) | 0 (0.00%) | 0 (0.00%) | 0 (0.00%) | 2 (2.90%) |
| Ocular trauma | 7 (11.67%) | 2 (5.00%) | 5 (21.74%) | 0 (0.00%) | 6 (26.62%) | 1 (0.03%) | 6 (9.09%) | 1 (1.45%) |
| Age-related macular degeneration | 2 (3.33%) | 1 (2.50%) | 0 (0.00%) | 0 (0.00%) | 2 (0.09%) | 1 (0.03%) | 0 (0.00%) | 0 (0.00%) |
| Optic neuropathy | 2 (3.33%) | 4 (16.00%) | 2 (8.70%) | 0 (0.00%) | 2 (0.09%) | 3 (0.10%) | 2 (3.03%) | 1 (1.45%) |
| Corneal opacity | 1 (1.67%) | 0 (0.00%) | 0 (0.00%) | 0 (0.00%) | 1 (0.04%) | 0 (0.00%) | 0 (0.00%) | 0 (0.00%) |
| Uveitis | 0 (0.00%) | 0 (0.00%) | 0 (0.00%) | 0 (0.00%) | 0 (0.00%) | 0 (0.00%) | 0 (0.00%) | 0 (0.00%) |
| Myopic retinopathy | 0 (0.00%) | 0 (0.00%) | 0 (0.00%) | 0 (0.00%) | 0 (0.00%) | 0 (0.00%) | 0 (0.00%) | 0 (0.00%) |
| Other | 37 (61.67%) | 21 (52.50%) | 13 (56.52%) | 9 (64.29%) | 42 (1.86%) | 20 (0.68%) | 25 (37.88%) | 18 (26.09%) |
| Total eyes | 66 (100%) | 40 (100%) | 23 (100%) | 14 (100%) | 2254 (100%) | 2938 (100%) | 66 (100%) | 69 (100%) |

Abbreviations: NA, not applicable; VI, visual impairment; WHO, World Health Organization.

# **Supplementary Table 9.** Associations between risk factors and the prevalence of binocular visual impairment by multiple logistic regression analysis (using the WHO criteria).

| Risk factors | Visual impairment based on BCVA | | | | | Blindness based on BCVA | | | | |
| --- | --- | --- | --- | --- | --- | --- | --- | --- | --- | --- |
|  | OR | Std. Err | Z | P | 95% CI | OR | Std. Err | Z | P | 95% CI |
| Hypertension | 2.12 | 0.40 | 3.92 | 0.000 | 1.45-3.08 | 1.01 | 0.66 | 0.01 | 0.990 | 0.28-3.61 |
| Diabetes mellitus | 1.83 | 0.43 | 2.54 | 0.011 | 1.15-2.91 | 4.96 | 2.80 | 2.83 | 0.010 | 1.63-15.02 |
| Hyperlipidemia | 0.59 | 0.22 | -1.43 | 0.154 | 0.28-1.22 | 1(omitted) | | | | |
| Coronary heart disease | 1(omitted) | | | | | 1(omitted) | | | | |
| Stroke | 1(omitted) | | | | | 1(omitted) | | | | |

Abbreviations: OR, odds ratios; Std.Err, standard error; CI, confidence interval.

**Supplementary Table 10.** Prevalence of hyperopia, myopia and anisometropia (N=13208).

|  | Number of participants | Prevalence (%) | 95%CI |
| --- | --- | --- | --- |
| Hyperopia | 3294 | 24.94 | 24.21-25.68 |
| Mild hyperopia | 3160 | 23.92 | 23.20-24.66 |
| Moderate hyperopia | 105 | 0.79 | 0.66-0.96 |
| High hyperopia | 29 | 0.22 | 0.15-0.32 |
| Emmetropia | 2682 | 20.31 | 19.63-21.00 |
| Myopia | 7232 | 54.75 | 53.90-55.60 |
| Mild myopia | 3861 | 29.23 | 28.46-30.01 |
| Moderate myopia | 2174 | 16.46 | 15.84-17.10 |
| High myopia | 1197 | 9.06 | 8.58-9.56 |
| Anisometropia | 1484 | 11.24 | 10.71-11.79 |

# **Supplementary Table 11.** Age-specific and gender-specific prevalence of hyperopia, myopia, emmetropia and anisometropia.

| Age group | Gender | Hyperopia | | | Emmetropia | | | High myopia | | | Myopia | | | Anisometropia | | |
| --- | --- | --- | --- | --- | --- | --- | --- | --- | --- | --- | --- | --- | --- | --- | --- | --- |
|  |  | No. | % | 95%CI | No. | % | 95%CI | No. | % | 95%CI | No. | % | 95%CI | No. | % | 95%CI |
| 18-24 | Male | 40 | 3.07 | 2.26-4.16 | 162 | 12.43 | 10.75-14.34 | 206 | 15.81 | 13.93-17.89 | 1101 | 84.50 | 82.43-86.36 | 215 | 16.50 | 14.58-18.62 |
|  | Female | 21 | 1.90 | 1.24-2.89 | 40 | 3.61 | 2.66-4.89 | 269 | 24.28 | 21.84-26.89 | 1047 | 94.49 | 92.99-95.69 | 199 | 17.96 | 15.81-20.33 |
|  | Male ＆Female | 61 | 2.53 | 1.97-3.24 | 202 | 8.38 | 7.34-9.55 | 475 | 19.70 | 18.16-21.34 | 2148 | 89.09 | 87.78-90.28 | 414 | 17.17 | 15.72-18.73 |
| 25-34 | Male | 14 | 1.83 | 1.08-3.06 | 59 | 7.70 | 6.01-9.82 | 126 | 16.45 | 13.99-19.25 | 693 | 90.47 | 88.18-92.36 | 101 | 13.19 | 10.97-15.77 |
|  | Female | 16 | 1.47 | 0.90-2.38 | 76 | 6.97 | 5.60-8.64 | 182 | 16.68 | 14.58-19.01 | 999 | 91.57 | 89.76-93.08 | 137 | 12.56 | 10.72-14.66 |
|  | Male ＆Female | 30 | 1.62 | 1.13-2.30 | 135 | 7.27 | 6.17-8.54 | 308 | 16.59 | 14.96-18.35 | 1692 | 91.11 | 89.73-92.33 | 238 | 12.82 | 11.37-14.42 |
| 35-44 | Male | 21 | 3.52 | 2.31-5.35 | 100 | 16.78 | 13.99-20.00 | 63 | 10.57 | 8.34-13.31 | 475 | 79.70 | 76.27-82.74 | 60 | 10.07 | 7.89-12.76 |
|  | Female | 27 | 2.88 | 1.98-4.17 | 168 | 17.91 | 15.59-20.50 | 82 | 8.74 | 7.09-10.73 | 743 | 79.21 | 76.49-81.69 | 80 | 8.53 | 6.90-10.50 |
|  | Male ＆Female | 48 | 3.13 | 2.37-4.13 | 268 | 17.47 | 15.65-19.45 | 145 | 9.45 | 8.09-11.02 | 1218 | 79.40 | 77.30-81.35 | 140 | 9.13 | 7.78-10.67 |
| 45-54 | Male | 105 | 13.22 | 11.04-15.77 | 286 | 36.02 | 32.75-39.42 | 56 | 7.05 | 5.47-9.06 | 403 | 50.76 | 47.28-54.23 | 48 | 6.05 | 4.58-7.93 |
|  | Female | 223 | 18.41 | 16.33-20.70 | 442 | 36.50 | 33.83-39.25 | 75 | 6.19 | 4.97-7.70 | 546 | 45.09 | 42.30-47.90 | 62 | 5.12 | 4.01-6.51 |
|  | Male ＆Female | 328 | 16.36 | 14.80-18.04 | 728 | 36.31 | 34.23-38.44 | 131 | 6.53 | 5.53-7.70 | 949 | 47.33 | 45.15-49.52 | 110 | 5.49 | 4.57-6.57 |
| 55-64 | Male | 453 | 45.03 | 41.98-48.12 | 301 | 29.92 | 27.17-32.83 | 30 | 2.98 | 2.09-4.23 | 252 | 25.05 | 22.47-27.82 | 79 | 7.85 | 6.34-9.69 |
|  | Female | 757 | 49.87 | 47.35-52.38 | 407 | 26.81 | 24.64-29.10 | 41 | 2.70 | 1.99-3.65 | 354 | 23.32 | 21.26-25.52 | 89 | 5.86 | 4.79-7.16 |
|  | Male ＆Female | 1210 | 47.94 | 45.99-49.89 | 708 | 28.05 | 26.33-29.84 | 71 | 2.81 | 2.23-3.54 | 606 | 24.01 | 22.38-25.72 | 168 | 6.66 | 5.75-7.70 |
| 65-74 | Male | 519 | 57.41 | 54.16-60.60 | 224 | 24.78 | 22.07-27.70 | 15 | 1.66 | 1.00-2.73 | 161 | 17.81 | 15.45-20.44 | 113 | 12.50 | 10.50-14.82 |
|  | Female | 788 | 62.74 | 60.03-65.37 | 246 | 19.59 | 17.48-21.88 | 26 | 2.07 | 1.41-3.02 | 222 | 17.68 | 15.66-19.89 | 145 | 11.54 | 9.89-13.43 |
|  | Male ＆Female | 1307 | 60.51 | 58.43-62.55 | 470 | 21.76 | 20.07-23.55 | 41 | 1.90 | 1.40-2.57 | 383 | 17.73 | 16.18-19.40 | 258 | 11.94 | 10.64-13.38 |
| 75-84 | Male | 119 | 40.89 | 35.38-46.65 | 76 | 26.12 | 21.38-31.48 | 14 | 4.81 | 2.87-7.97 | 96 | 32.99 | 27.82-38.61 | 60 | 20.62 | 16.35-25.66 |
|  | Female | 170 | 47.49 | 42.35-52.68 | 74 | 20.67 | 16.78-25.19 | 9 | 2.51 | 1.31-4.76 | 114 | 31.84 | 27.22-36.86 | 81 | 22.63 | 18.58-27.26 |
|  | Male ＆Female | 289 | 44.53 | 40.74-48.38 | 150 | 23.11 | 20.03-26.52 | 23 | 3.54 | 2.37-5.28 | 210 | 32.36 | 28.86-36.06 | 141 | 21.73 | 18.72-25.07 |
| ≥85 | Male | 11 | 28.95 | 16.68-45.33 | 10 | 26.32 | 14.66-42.61 | 2 | 5.26 | 1.30-19.04 | 17 | 44.74 | 29.75-60.75 | 9 | 23.68 | 12.70-39.84 |
|  | Female | 10 | 33.33 | 18.77-51.97 | 11 | 36.67 | 21.38-55.20 | 1 | 3.33 | 0.45-20.76 | 9 | 30.00 | 16.22-48.68 | 6 | 20.00 | 9.14-38.31 |
|  | Male ＆Female | 21 | 30.88 | 21.02-42.87 | 21 | 30.88 | 21.02-42.87 | 3 | 4.41 | 1.42-12.90 | 26 | 38.24 | 27.44-50.33 | 15 | 22.06 | 13.71-33.52 |
| Gender-specific differences | | χ^2^=31.88 | | *P*<0.001 | χ^2^=7.09 | | *P*=0.008 | χ^2^=0.07 | | *P*=0.788 | χ^2^=7.60 | | *P*=0.006 | χ^2^=6.21 | | *P*=0.013 |

Abbreviations: No, number.

# **Supplementary Table 12.** Factors associated with the prevalence of hyperopia, myopia and high myopia by multiple logistic regression analysis.

| Associated factors | Hyperopia | | | Myopia | | | High myopia | | |
| --- | --- | --- | --- | --- | --- | --- | --- | --- | --- |
|  | OR | P | 95% CI | OR | P | 95% CI | OR | P | 95% CI |
| Sex (women) | 0.98 | 0.657 | 0.89-1.08 | 1.16 | 0.001 | 1.07-1.27 | 1.25 | <0.001 | 1.11-1.41 |
| Age group | 2.10 | <0.001 | 2.02-2.18 | 0.59 | <0.001 | 0.57-0.61 | 0.91 | <0.001 | 0.86-0.95 |
| BMI (>24) | 1.07 | 0.149 | 0.98-1.18 | 0.92 | 0.069 | 0.84-1.01 | 0.93 | 0.268 | 0.81-1.06 |
| Education | 0.66 | <0.001 | 0.64-0.69 | 1.71 | <0.001 | 1.65-1.78 | 1.81 | <0.001 | 1.70-1.93 |
| Economic status | 0.94 | 0.134 | 0.87-1.02 | 0.92 | 0.027 | 0.86-0.99 | 0.77 | <0.001 | 0.71-0.83 |

Abbreviations: OR, odds ratios; CI, confidence interval.

**Supplementary Table 13.** Comparison of the prevalence and causes of binocular visual impairment and blindness based on presenting visual acuity in population with all ages (Using the WHO criteria)

| Year | District | Causes of binocular visual impairment (Proportion %) | | | Causes of binocular blindness (Proportion %) | | | Prevalence (%) | |
| --- | --- | --- | --- | --- | --- | --- | --- | --- | --- |
|  |  | 1 ^st^ | 2 ^nd^ | 3 ^rd^ | 1 ^st^ | 2 ^nd^ | 3 ^rd^ | Visual impairment | Blindness |
| 1990^17,18^ | Glob | URE  (53.03%) | Cataract  (24.79%) | Macular degeneration  (5.42%) | Cataract  (35.48%) | URE  (20.24%) | Glaucoma  (8.41%) | Moderate VI:3.11%  Severe VI:0.43%  MSVI: 3.83% | 0.75% |
| 2015^17,19^ | Glob | URE  (53.72%) | Cataract  (24.05%) | Macular degeneration  (4.00%) | Cataract  (33.47%) | URE  (20.62%) | Glaucoma  8.30%) | Moderate VI:3.15%  Severe VI:0.42%  MSVI: 2.95% | 0.49% |
| 2020^20^ | Glob | NA | NA | NA | NA | NA | NA | 3.58% | 0.53% |
| 1990^17,18^ | China | URE  (65.32%) | Cataract  (17.78%) | Macular degeneration  (3.39%) | Cataract  (33.57%) | URE  (14.20%) | Glaucoma  (6.75%) | Moderate VI:2.29%  Severe VI:0.24% | 0.64% |
| 2019^18^ | China | URE  (51.65%) | Cataract  (30.03%) | Macular degeneration  (4.51%) | Cataract  (33.92%) | URE  (13.19%) | Glaucoma  (5.57%) | Moderate VI:2.57%  Severe VI:0.25% | 0.48% |
| 2019^18^ | China, Jiangsu | URE  (52.09%) | Cataract  (34.38%) | Macular degeneration  (3.64%) | Cataract  (29.52%) | URE  (19.08%) | Macular degeneration  (6.74%) | Moderate VI: 2.5%–2.8%  Severe VI: 0.296–0.330% | 0.30%–0.45% |
| **2022** | **China, Jiangsu** | **URE**  **(93.40%)** | **Cataract**  **(1.31%)** | **Amblyopia**  **(0.48%)** | **URE**  **(50.79%)** | **Cataract**  **(12.70%)** | **Amblyopia**  **(4.76%)** | **21.04%** | **0.47 %** |

Results of this study were highlighted in bold font. Abbreviations: NA, non applicable; URE, uncorrected refractive error.

**Supplementary Table 14.** Comparison of the distribution of spherical equivalent refraction in population with all ages.

|  |  | Mild hyperopia | Moderate hyperopia | High hyperopia | Emmetropia | Myopia | Mild myopia | Moderate myopia | High myopia | Anisometropia |
| --- | --- | --- | --- | --- | --- | --- | --- | --- | --- | --- |
| 1990-2013 | Europe^21^ | NA | NA | 5.37% | NA | 30.60% | 19.50% | 8.08% | 2.71% | NA |
| 2015-2019 | Ireland^22^ | NA | NA | NA | 27.13% | 32.75% | NA | NA | 2.38% | 13.42% |
| 2007 | Japan^23^ | NA | NA | NA | NA | 41.80% | NA | NA | 8.20% | 15.10% |
| 2001 | China, Beijing^24^ | NA | NA | NA | NA | 22.90% | NA | NA | 16.90% | NA |
| **2022** | **China, Jiangsu** | **23.92%** | **0.79%** | **0.22%** | **20.31%** | **54.75%** | **29.23%** | **16.46%** | **9.06%** | **11.24%** |

Results of this study were highlighted in bold font. Abbreviations: NA, non applicable.

**Supplementary Table 15.** Comparison of the binocular visual impairment and blindness causes in Chinese and global populations aged ≥50 years old (Using the WHO criteria).

|  | Year | District | Causes of binocular visual impairment (Proportion %) | | | Causes of binocular blindness (Proportion %) | | | Prevalence (%) | |
| --- | --- | --- | --- | --- | --- | --- | --- | --- | --- | --- |
|  |  |  | 1 ^st^ | 2 ^nd^ | 3 ^rd^ | 1 ^st^ | 2 ^nd^ | 3 ^rd^ | VI | Blindness |
| Presenting VA (WHO criteria) | 1990 ^17,18^ | Glob | URE  (50.80%) | Cataract  (26.62%) | Macular degeneration  (5.97%) | Cataract  (36.67%) | URE  (19.58%) | Glaucoma  (8.66%) | 13.08% | 2.84% |
|  | 2015^17,19^ | Glob | URE  (52.34%) | Cataract  (24.75%) | Macular degeneration  (4.16%) | Cataract  (35.15%) | URE  (20.28%) | Glaucoma  (8.49%) | 10.41% | 1.90% |
|  | 2020^17^ | Glob | URE  (52.61%) | Cataract  (25.15%) | Macular degeneration  (4.38%) | Cataract  (34.73%) | URE  (20.58%) | Glaucoma  (8.43%) | NA | NA |
|  | 2020^17^ | East Asia | URE  (47.26%) | Cataract  (32.61%) | Macular degeneration  (1.60%) | Cataract  (43.50%) | URE  (12.93%) | Glaucoma  (7.11%) | NA | NA |
|  | 2010-2013^25^ | Chinese American | URE  (55.40%) | Cataract  (12.30%) | Myopic retinopathy (10.80%) | Myopic retinopathy (33.33%) | Glaucoma  (33.33%) | Optic neuropathy (33.33%) | 0.50% | 0.03% |
|  | 1990^18^ | China | URE  (65.32%) | Cataract  (17.78%) | Macular degeneration  (3.39%) | Cataract  (33.57%) | URE  (14.20%) | Glaucoma  (6.75%) | NA | NA |
|  | 2006^1^ | China | Cataract  (13.60%) | URE  (13.20%) | Retinal diseases (2.25%) | NA | NA | NA | 31.70% | 2.29% |
|  | 2014^1^ | China | URE  (15.80%) | Cataract  (11.40%) | Retinal diseases (2.81%) | NA | NA | NA | 32.40% | 1.66% |
|  | 2019^18^ | China | URE  (51.65%) | Cataract  (30.03%) | Macular degeneration  (4.51%) | Cataract  (33.92%) | URE  (13.19%) | Glaucoma  (5.57%) | NA | NA |
|  | 2003-2004^4^ | China，Guangzhou | Cataract  (45.30%) | URE  (43.90%) | Glaucoma  (2.80%) | Cataract  (39.6%) | Glaucoma  (11.00%) | URE  (8.80%) | 10.10% | 0.60% |
|  | **2022** | **China, Jiangsu** | **URE**  **(93.61%)** | **Cataract**  **(1.25%)** | **Amblyopia**  **(0.46%)** | **URE**  **(50.00%)** | **Cataract**  **(12.90%)** | **Amblyopia**  **(0.46%)** | **10.13%** | **0.58%** |
| BCVA (WHO criteria)  20 | 2010-2013^25^ | American  Chinese | Cataract (27.60%) | Myopic retinopathy (24.10%) | Macular degeneration  (13.80%) | Myopic retinopathy (33.33%) | Glaucoma (33.33%) | Optic neuropathy (33.33%) | 0.20% | 0.02% |
|  | 1997-1998^26^ | Singaporean  Chinese | Cataract (58.82%) | NA | NA | Glaucoma  (60.00%) | Cataract  (20.00%) | Macular degeneration (20.00%) |  |  |
|  | 2003-2004^4^ | China，Guangzhou | Cataract (71.80%) | Macular degeneration  (7.30%) | Myopic retinopathy (6.50%) | Cataract (44.70%) | Glaucoma (11.80%) | Myopic retinopathy (7.90%) | 3.10% | 0.50% |
|  | 2012-2013^2^ | China, Jiangsu | Cataract (59.10%) | Myopic retinopathy (17.60%) | Macular degeneration  (11.6%) | Cataract (48.50%) | Myopic retinopathy (17.20%) | Macular degeneration  (10.10%) | 5.10% | 1.00% |
|  | **2022** | **China, Jiangsu** | **Cataract**  **(16.92%)** | **Amblyopia**  **(8.46%)** | **Myopic retinopathy (3.85%)** | **Cataract**  **(20.00%)** | **Amblyopia**  **(20.00%)** | **Diabetic retinopathy**  **(13.33%)** | **1.88%** | **0.20%** |

Results of this study were highlighted in bold font. Abbreviations: NA, non applicable; URE, uncorrected refractive error; VI, visual impairme

**
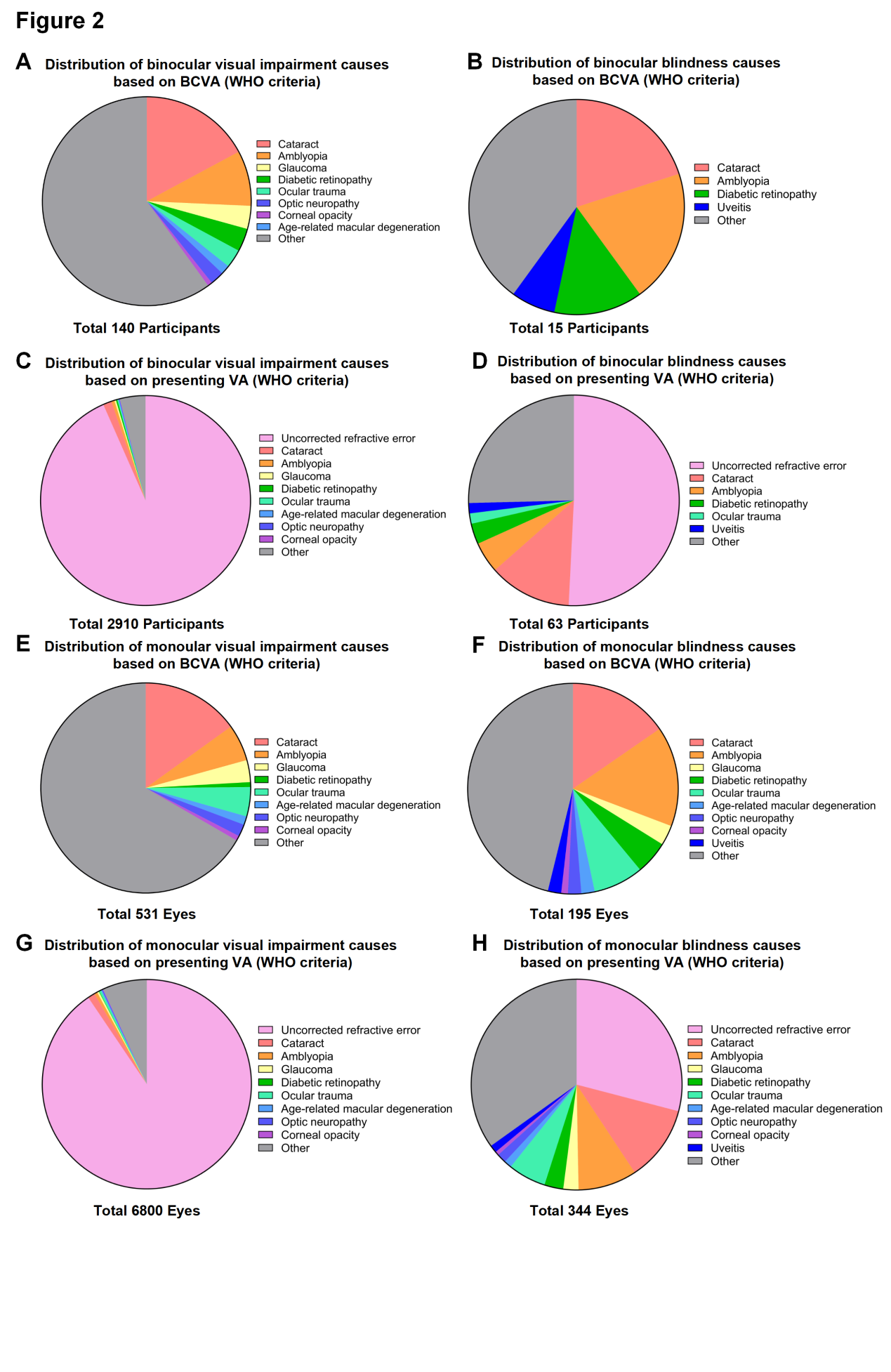
**

**Supplementary Figure 1.** Pie charts depicting the distribution of visual impairment (VI) and blindness causes using the WHO criteria. **(a-b)** binocular VI and blindness causes based on best-corrected visual acuity (BCVA). **(c-d)** binocular VI and blindness causes based on presenting visual acuity (VA). **(e-f)** Monocular VI and blindness causes based on best-corrected visual acuity (BCVA). **(g-h)** Monocular VI and blindness causes based on presenting visual acuity (VA).

**References**

1 Zhao, J. *et al.* Causes of Visual Impairment and Blindness in the 2006 and 2014 Nine-Province Surveys in Rural China. *Am. J. Ophthalmol.* **197,** 80-87 (2019).

2 Tang, Y. *et al.* Prevalence and Causes of Visual Impairment in a Chinese Adult Population: The Taizhou Eye Study. *Ophthalmology*. **122,** 1480-1488 (2015).

3 Zhao, J. *et al.* Prevalence of vision impairment in older adults in rural China: the China Nine-Province Survey. *Ophthalmology*. **117,** 409-416, 416.e401 (2010).

4 Huang, S., Zheng, Y., Foster, P., Huang, W., He, M. Prevalence and causes of visual impairment in Chinese adults in urban southern China. *Archives of ophthalmology (Chicago, Ill. : 1960)*. **127,** 1362-1367 (2009).

5 Varma, R., Wang, M., Ying-Lai, M., Donofrio, J., Azen, S. The prevalence and risk indicators of uncorrected refractive error and unmet refractive need in Latinos: the Los AngelesLatino Eye Study. *Invest Ophthalmol Vis Sci*. **49,** 5264-5273 (2008).

6 Zhou, Y., Li, G., Li, H. Automatic Cataract Classification Using Deep Neural Network With Discrete State Transition. *IEEE transactions on medical imaging*. **39,** 436-446 (2020).

7 Wang, Y. *et al.* Prevalence and causes of amblyopia in a rural adult population of Chinese the Handan Eye Study. *Ophthalmology*. **118,** 279-283 (2011).

8 Foster, P., Buhrmann, R., Quigley, H., Johnson, G. The definition and classification of glaucoma in prevalence surveys. *Br J Ophthalmol*. **86,** 238-242 (2002).

9 Kuang, T. *et al.* Distribution and associated factors of optic disc diameter and cup-to-disc ratio in an elderly Chinese population. *Journal of the Chinese Medical Association : JCMA*. **77,** 203-208 (2014).

10 Diabetic Retinopathy Group of Chinese Diabetes Society. Chinese multidisciplinary expert consensus on the prevention and treatment of diabetic eye disease (2021 edition). *Chinese Journal of Diabetes Mellitus*. **13,** 1026-1042 (2021).

11 Yin, L., Zhang, D., Ren, Q., Su, X.Sun, Z. Prevalence and risk factors of diabetic retinopathy in diabetic patients: A community based cross-sectional study. *Medicine (Baltimore)*. **99,** e19236 (2020).

12 Zhou, J., Wang, F., Lu, H., Liang, Y., Wang, N. Ocular Trauma in a Rural Population of North China: The Handan Eye Study. *Biomedical and environmental sciences : BES*. **28,** 495-501 (2015).

13 Chua, D. *et al.* The prevalence and risk factors of ocular trauma: the Singapore Indian eye study. *Ophthalmic Epidemiol.* **18,** 281-287 (2011).

14 Wong, M. *et al.* Prevalence, subtypes, severity and determinants of ocular trauma: The Singapore Chinese Eye Study. *Br J Ophthalmol*. **102,** 204-209 (2018).

15 Klein, R. *et al.* The Wisconsin age-related maculopathy grading system. *Ophthalmology*. **98,** 1128-1134 (1991).

16 Sparrow, J., Dickinson, A., Duke, A. The Wisconsin Age-related Macular Degeneration grading system: performance in an independent centre. *Ophthalmic Epidemiol.* **4,** 49-55 (1997).

17 Flaxman, S. *et al.* Global causes of blindness and distance vision impairment 1990-2020: a systematic review and meta-analysis. *The Lancet. Global health*. **5,** e1221-e1234 (2017).

18 Xu, T. *et al.* Prevalence and causes of vision loss in China from 1990 to 2019: findings from the Global Burden of Disease Study 2019. *The Lancet. Public health*. **5,** e682-e691 (2020).

19 Bourne, R. *et al.* Magnitude, temporal trends, and projections of the global prevalence of blindness and distance and near vision impairment: a systematic review and meta-analysis. *The Lancet. Global health*. **5,** e888-e897 (2017).

20 GBD 2019 Blindness and Vision Impairment Collaborators, Vision Loss Expert Group of the Global Burden of Disease Study. Trends in prevalence of blindness and distance and near vision impairment over 30 years: an analysis for the Global Burden of Disease Study. *The Lancet. Global health*. **9,** e130-e143 (2021).

21 Williams, K. *et al.* Prevalence of refractive error in Europe: the European Eye Epidemiology (E(3)) Consortium. *Eur. J. Epidemiol.* **30,** 305-315 (2015).

22 Longwill, S., Moore, M., Flitcroft, D., Loughman, J. Using electronic medical record data to establish and monitor the distribution of refractive errors. *Journal of optometry***,** S32-S42 (2022).

23 Akira Sawada A. T., Makoto Araie, Aiko Iwase, Tetsuya Yamamoto, Tajimi Study Group. Refractive Errors in an Elderly Japanese Population: The Tajimi Study. *Ophthalmology*. **115,** 363-370.e363 (2008).

24 Liang, Xu J. L., Tongtong, C., Ailian H., Guizhi, F., Rongxiu, Z., Hua, Y, Baochen, S., Jost B. J. Refractive Error in Urban and Rural Adult Chinese in Beijing. *Ophthalmology*. **112,** 1676-1683 (2005).

25 Varma, R. *et al.* Prevalence and Causes of Visual Impairment and Blindness in Chinese American Adults: The Chinese American Eye Study. *JAMA Ophthalmol*. **134,** 785-793 (2016).

26 Saw, S., Foster, P., Gazzard, G., Seah, S. Causes of blindness, low vision, and questionnaire-assessed poor visual function in Singaporean Chinese adults: The Tanjong Pagar Survey. *Ophthalmology*. **111,** 1161-1168 (2004).
